# Inhibition of GRK2-PDE4D Axis Suppresses Fibroblast-Like Synoviocytes Hyperplasia and Alleviates Experimental Arthritis

**DOI:** 10.7150/ijbs.100176

**Published:** 2025-01-27

**Authors:** Dafei Han, Hanfei Sun, Renhao Zhang, Hui Ge, Paipai Guo, Rui Chu, Ruhong Fang, Yongsheng Han, Shufang He, Rui Li, Jiajie Tu, Wei Wei, Yang Ma, Qingtong Wang

**Affiliations:** 1 Institute of Clinical Pharmacology, Anhui Medical University, Key Laboratory of Anti-Inflammatory and Immune Medicine, Ministry of Education, Anhui Collaborative Innovation Center of Anti-Inflammatory and Immune Medicine, Hefei, 230032, China.; 2 Department of Emergency Medicine, The First Affiliated Hospital of USTC, Division of Life Sciences and Medicine, University of Science and Technology of China, Hefei, 230001, China.; 3 Department of Anesthesiology and Perioperative Medicine, the Second Hospital of Anhui Medical University, Hefei 230601, China.; 4 The Third Affiliated Hospital of Anhui Medical University (The First People's Hospital of Hefei), Hefei 230061, China.

**Keywords:** Rheumatoid arthritis, Fibroblast-like synoviocytes, Phosphodiesterase 4D, G protein coupled receptor kinase 2, CP-25

## Abstract

PDE4D has been reported to exhibit significantly elevated levels in the synovium of RA patients compared with OA, yet its role in RA remains underexplored. This study aimed to elucidate the role of the GRK2-PDE4D axis in FLSs and explore its potential as a therapeutic target for RA. Abundant expression of both PDE4D and GRK2 was observed in synovial tissues from both experimental arthritis animals and RA patients, with synchronized expression noted in RA patients. Global deletion of *Pde4d* reduced disease incidence and alleviated arthritis in CIA mice. TNF-α upregulated PDE4D expression, causing abnormal FLSs activation and hyperproliferation. Inhibiting PDE4D restored cAMP levels, thereby reducing FLSs hyperproliferation, migration, and anti-apoptosis. Mechanistically, TNF-α-induced PDE4D upregulation was dependent on GRK2. Inhibition of GRK2 with CP-25, an esterification modification of paeoniflorin, reduced PDE4D expression and FLSs proliferation, while restoring cAMP levels. Both genetic deficiency and pharmacological inhibition of GRK2 decreased PDE4D expression, ameliorating arthritis severity in animal models. This is the first study to investigate the role of PDE4D in RA and to clarify that it can be regulated by GRK2. These findings suggest that targeting the GRK2-PDE4D axis represents a promising therapeutic strategy for RA.

## Introduction

Rheumatoid arthritis (RA), a widespread autoimmune disease, involves persistent synovial inflammation and progressive joint destruction, affecting approximately 1% of the global population^1^. Fibroblast-like synoviocytes (FLSs) play a crucial role in RA progression due to their hyperproliferation and invasiveness.

Cyclic adenosine monophosphate (cAMP), produced through the Gαs-cAMP signaling pathway, exerts potent immunomodulatory effects on immune responses^2^. Elevated intracellular cAMP inhibits T cell activation and suppresses the differentiation of monocytes^2^, ^3^. Moreover, an increased intracellular cAMP content effectively inhibits FLSs proliferation through diverse signaling pathways^4^. In previous studies, we observed decreased intracellular cAMP concentration in FLSs within experimental arthritis models^4^, ^5^. However, inhibiting G protein-coupled receptors (GPCRs) desensitization mediated by G protein coupled receptor kinase 2 (GRK2) led to increased cAMP concentration and relieved experimental arthritis models^4^. Therefore, agents enhancing cAMP production or suppressing cAMP hydrolysis are potential therapeutic strategies for inhibiting hyperproliferative FLSs in RA.

Intracellular cAMP is synthesized and hydrolyzed by adenylate cyclases and phosphodiesterases (PDEs), respectively. PDE4, a major cAMP-hydrolyzing isoenzymes, is ubiquitously expressed in both immune and non-immune cells^2^, ^6^. Inhibiting PDE4 effectively elevates intracellular cAMP concentration and inhibits various inflammatory responses, including proliferation, chemotaxis, and the secretion of inflammatory factors^2^. Apremilast, a novel oral PDE4 inhibitor approved for psoriasis and psoriasis arthritis, has been shown to inhibit TNF-α production from RA-FLSs, reduce RA-FLSs migration and invasion into cartilage, and ameliorate experimental arthritis models^7^, ^8^. Regrettably, most of PDE4 inhibitors have been discontinued due to severe side effects, including nausea, vomiting, diarrhea, and abdominal pain^2^. PDE4 is encoded by four separate genes *(PDE4A-D)*, and the catalytic domain is highly conserved among the four subtypes^2^. PDE4A, PDE4B, and PDE4D are ubiquitously expressed in various immune cells (T and B cells, dendritic cells, monocytes, macrophages eosinophils, neutrophils) and non-immune cells (FLSs, bronchial smooth muscle cells, chondrocytes), while PDE4C is minimally active or absent^2^, ^6^, ^9^-^11^. However, others have indicated that, although PDE4C is largely absent in immune cells, it is present in non-immune cells such as keratinocytes and epithelial cells^12^, ^13^. Among these, PDE4D has been implicated in multiple inflammatory diseases, and its inhibition can alleviate animal models of these diseases. For example, inhibition of PDE4D is a potential therapeutic approach for psoriasis^14^, ^15^. In a mouse model of asthma, the expression of PDE4D mRNA was increased, and this increased expression was significantly correlated with airway hyperreactivity. Treatment with ciclamilast, a PDE4 inhibitor, inhibited PDE4D expression, down-regulated cAMP-PDE4 activity, and reduced inflammatory cytokines^16^. The expression levels of PDE4D have been reported to be notably higher in the synovium and TNF-α-treated FLSs of RA patients^6^, suggesting that PDE4D may be an effective target for RA treatment, particularly in the context of hyperproliferative FLSs. However, the effect of PDE4D in RA, a chronic inflammatory disease, has not yet been studied. It is worth noting that there are no marketed PDE4D subtype-selective inhibitors, and BPN14770, an allosteric inhibitor of PDE4D, is being studied in the context of cognitive function and other brain disorders, but still exhibits some inevitable side effects^17^, ^18^. Therefore, investigating the mechanisms behind PDE4D overexpression and exploring strategies to manipulate its expression in FLSs are crucial for advancing RA treatment.

GRK2 is implicated in various diseases, including RA, through its regulation of the desensitization and internalization of multiple GPCRs^19^. We have previously reported elevated GRK2 expression in synovial tissues of both RA patients and arthritis animal models^20^, ^21^. The increased GRK2 results in GPCRs desensitization and subsequent hyperplasia of FLSs, thereby driving the irreversible progression of RA^4^. Additionally, besides GPCRs desensitization, GRK2 influences key signaling pathways, including extracellular signal-regulated kinase 1/2 (ERK1/2), phosphatidylinositol 3-kinase (PI3K), mitogen-activated protein kinase (MAPK), and nuclear factor kappa-B (NF-κB)^22^. However, the functional relevance and the underlying mechanisms of GRK2-regulated PDE4D in RA have yet to be explored. Based on the findings, we hypothesize that in RA-FLSs, increased GRK2 results in PDE4D upregulation, reducing intracellular cAMP and exacerbating RA by promoting uncontrolled FLSs hyperproliferation. Paeoniflorin-6′-O-benzene sulfonate, designated as CP-25 (chemical formula: C_29_H_32_O_13_S; Chinese patent number: ZL201210030616.4), is an esterified derivative of Paeoniflorin developed within our laboratory. As an inhibitor of GRK2, it displays a diverse range of anti-inflammatory and immunomodulatory activities in autoimmune diseases, including systemic lupus erythematosus, inflammatory bowel disease, Sjogren's syndrome and RA^23^. Studies have demonstrated that CP-25 suppresses the abnormal proliferation of FLSs mediated by EP4 desensitization via GRK2^24^. However, the potential of CP-25 to regulate PDE4D remains unexplored. In the present study, we investigated, for the first time, the impact of CP-25 on PDE4D through GRK2.

In this study, we demonstrated a significantly upregulation of PDE4D in FLSs from both RA patients and experimental arthritis models, which contributes to the hyperproliferation of FLSs. Genetic deficiency of *Pde4d* reduced arthritis severity in CIA mice. Furthermore, we found that the heightened expression of PDE4D was regulated by GRK2. Genetic deletion or pharmacological inhibition of GRK2 significantly attenuated the progression of arthritis in animal models. This is the first study to evaluate the role of PDE4D and GRK2-PDE4D axis in RA by suppressing FLSs proliferation, thereby providing an alternative therapeutic approach for RA.

## Materials and Methods

### Animal

*Pde4d^+/-^* mice (Strain NO. T032409) and *Grk2^+/-^* mice (Strain NO. T014508) were purchased from GemPharmatech (Nanjing, China). Male Sprague Dawley rats, 6-8-weeks-old, (150 ~ 200 g) were purchased from Charles River Laboratories (Beijing, China). The animals were housed in a specific pathogen-free animal laboratory at the institute of Clinical Pharmacology of Anhui Medical University. All animal experiment protocols were reviewed and approved by the Ethics Committee of Institute of Clinical Pharmacology of Anhui Medical University (LLSC20190307).

### Human samples

Knee synovium tissues used in this investigation were obtained from RA and OA patients undergoing knee arthroplasty at the First Affiliated Hospital of Anhui Medical University. The healthy control synovium tissues were obtained from trauma patients without inflammatory knee joint disorders. Each patient gave written informed consent and the research protocols were approved by the Ethics Committee of Institute of Clinical Pharmacology of Anhui Medical University (Ethical NO. 20200608).

### Primary FLSs isolation, culture, transfection and treatment

Primary human and rat-FLSs were obtained from fresh synovium tissues as previously described^4^, and cultured in Dulbecco's modified Eagle medium with 20% fetal bovine serum (FBS) and 1% penicillin-streptomycin. Cells were transfection with GRK2 siRNA and negative control (NC) siRNA, GRK2 overexpressed plasmid and vector, or pcDNA3-ICUE3 using Lipofectamine 2000 and P300 (11668027, Thermo Fisher Scientific, Waltham, MA, USA) according to the manufacturer's instructions. The siRNA and NC sequences used for transfection are listed in Table S1.

To elucidate the intracellular pathway contributing to GRK2-mediated PDE4D regulation, the rat-FLSs were divided into different groups (blank, TNF-α alone, and TNF-α + different inhibitors as indicated). The inhibitors included an NF-κB inhibitor (JSH-23, 10 μM, HY-13982), a PI3K inhibitor (NVP-BAG956, 10 μM, HY-13333), a JNK inhibitor (Bentamapimod, 7.5 μM, HY-14761), a p38 inhibitor (Doramapimod, 10 μM, HY-10320), and an ERK inhibitor (Ravoxertinib, 5 μM, HY-15947), all obtained from MedChemExpress (NJ, USA). CP-25 (1 μM) was used as a positive control to inhibit PDE4D expression.

### Quantitative PCR (qPCR) and PCR for genotyping

Total RNA from FLSs of CIA and normal rats was extracted using Trizol (15596-018, Thermo Fisher Scientific, MA, USA) following the manufacturer's instructions. Complementary DNA synthesis was performed using 1 μg of RNA with MMLV Reverse Transcriptase (R122-01, Vazyme, Jiangsu, China) following the manufacturer's protocols. SYBR Green Master Mix (Q111-02, Vazyme, Jiangsu, China) was used for qPCR, and gene expression was normalized to Gapdh using the 2^-ΔΔCt^ method. The specific samples for PCR genotyping experiments were collected from the tails of littermate mice. DNA was extracted from approximately 3 mm long tail tips of littermates using alkaline lysis and centrifugation. PCR amplification was then performed using the extracted DNA and corresponding primers (T100 Thermal Cycler, Bio-Rad, CA, USA). The amplified products were separated by 2% agarose gel electrophoresis. Primer sequences for qPCR and genotyping are list in Table S1.

### Hematoxylin and eosin (H&E) and Safranin O-fast green (SO/FG) staining

Following euthanasia of mice and rats at specified times, joint synovial tissues and hind limbs from both species were fixed, decalcified, and embedded. Synovium specimens from RA and OA patients underwent fixation and embedding. Paraffin slides (5 μm) were dewaxed, rehydrated, and subjected to H&E or SO/FG staining. Scanning of slides was performed using Pannoramic MIDI Ⅱ (3DHISTECH, Budapest, Hungary), and analysis was carried out using CaseViewer software 2.4 (3DHISTECH, Budapest, Hungary). The histopathological changes were determined by evaluating the inflammation, bone erosion, pannus formation, and synoviocytes proliferation. Each parameter was graded from 0 to 4 (0 = no effect, 4 = severe effect) as previously described^4^, ^25^.

### Immunohistochemical (IHC) staining

IHC staining was performed as previously described^26^. Briefly, paraffin-embedded synovium tissues were sectioned, deparaffinised and hydrated routinely, followed by antigen retrieval. After neutralizing endogenous peroxidase, the slides were incubated overnight at 4 °C with anti-PDE4D and anti-GRK2 primary antibodies (see Table S2). The following day, biotin-labelled secondary antibody incubation was performed, followed by DAB staining and counterstaining with hematoxylin. Finally, the slides were mounted with neutral gum, scanned using Pannoramic MIDI Ⅱ (3DHISTECH, Budapest, Hungary) and analyzed via CaseViewer software 2.4 (3DHISTECH, Budapest, Hungary).

### Immunofluorescence (IF) staining

Paraffin-embedded synovium tissues were performed following the IHC protocol. For subsequent IF staining, slides were permeabilized and blocked with bovine serum albumin for 1 h at room temperature. Synovium slides were then incubated overnight at 4 °C with anti-PDE4D, anti-GRK2, anti-p65 and anti-p-ERK primary antibodies (detailed information was described in Table S2). After rewarming and washing, the slides were incubated with Alexa Fluor 488-Anti-mouse and Alexa Fluor 594-Anti-rabbit secondary antibodies (detailed information was described in Table S2) at room temperature for 1 h. Finally, the slides were mounted with neutral gum, scanned using Pannoramic MIDI Ⅱ (3DHISTECH, Budapest, Hungary) and analyzed via CaseViewer software 2.4 (3DHISTECH, Budapest, Hungary).

### Western blot assay

Proteins from FLSs, mouse tails, or paws were extracted using RIPA lysis buffer supplemented with a protease and phosphatase inhibitor cocktail (P1048, Beyotime, Shanghai, China) as previously described^26^. Briefly, protein lysates were separated on 10% SDS-PAGE gels, transferred onto PVDF membranes (IPVH00010, Millipore, USA), and blocked with 5% skim milk. Membranes were then incubated with primary antibodies overnight at 4 °C (detailed information was described in Table S2), followed by incubation with HRP-conjugated anti-rabbit or anti-mouse IgG secondary antibodies for 1 h at room temperature. Protein bands were visualized using enhanced chemiluminescence and band intensities (GAPDH as a loading control) were quantified suing ImageJ software 1.8.0 (NIH, MD, USA).

### Transwell assay

To assess the migration ability of RA-FLSs, a 24-well chamber (8-µm-pore) (Corning, Tewksbury, MA, USA) was used. Briefly, 5×10^3^ RA-FLSs in 250 μl of serum-free medium were seeded in the upper compartment of the transwell apparatus. To the lower chambers, 750 μl of complete medium containing 10% FBS was added. After 24 h of culturing, non-migrated FLSs on the upper surface of the chambers were gently removed, and the migratory cells on the lower surface were fixed and stained. Five random areas of each well were photographed using a microscope (OLYMPUS TH4-200, Japan) and counted by ImageJ software 1.8.0 (NIH, MD, USA).

### Apoptosis assay

Apoptotic FLSs were determined using the Annexin V-APC/PI Apoptosis Detection kit (BB-41033, Bestbio, Jiangsu, China) on a BD FACSCanto^TM^ flow cytometer following the manufacturers' protocols. Briefly, cells were collected, washed twice with cold PBS, incubated with Annexin V-APC and PI in 1× Annexin V binding solution, and then analyzed using FlowJo software 10.8.1 (BD Biosciences, NJ, USA) for data acquisition, analysis, and reporting.

### Cellular vitality and proliferation assay

To assess the cellular vitality of FLSs, pre-treated FLSs were plated at 5×10^3^ cells per well in a 96-well plate and cultured at 37 °C and 5% CO_2_ for 5 h. Subsequently, 10 µl of CCK-8 solution (BS350A, Biosharp, Hefei, China) was added to each well and incubated for an additional 5 h. T lymphocytes obtained from the thymus of mice were seeded in a 96-well plate (1×10^6^ per well) and stimulated with 5 mg/L ConA at 37 °C and 5% CO_2_ for 48 h. Four hours before the end of the culture, 10 μL of CCK-8 solution was added to each well and incubated for an additional 5 h. Cellular vitality, reflected by absorbance at OD 450 nm, was measured using a microplate reader (Infinite M1000 PRO, Tecan, Switzerland). For detecting FLSs cellular proliferation, 5×10^3^ FLSs were seeded into a 96-well plate, treated differently for 48 h, washed with cold PBS, fixed with 4% paraformaldehyde for 20 min, and incubated with DAPI for 5 min at room temperature. Cell numbers in per well were analyzed using a high content cell imaging system (HCCIS) (Image Xpress Micro 4, Molecular Devcies, CA, USA).

### Enzyme-linked immunosorbent assay (ELISA)

For IL-6 quantification, the supernatant from FLSs was collected, centrifuged to remove cellular debris, and immediately used for IL-6 quantification following the manufacturer's recommendations (EK106, Multi Science, Hangzhou, China). For cAMP quantification, FLSs were washed twice with cold PBS and lysed by 0.1 M HCl for 10 min at room temperature. After centrifugation, the supernatant was immediately assayed using a cAMP Enzyme Immunoassay kit (ZY-cAMP-HU, Zeye, Shanghai, Chian) following the manufacturer's indications. Animal serum was collected, and the level of TNF-α was detected using commercial mouse (E-EL-M3063) and rat (E-EL-R2856) TNF-α ELISA Kits (Elabscience, Wuhan, China), respectively, according to the manufacturer's instructions.

### FRET analysis of cAMP

For cAMP FRET analysis, rat-FLSs transfected with the pcDNA3-ICUE3 plasmid were treated with TNF-α (20 ng/ml) for 24 h or co-treated with TNF-α and CP-25 (1 μM) for 24 h. Cell slides were placed in a glass-bottom petri dish containing 2 ml PBS, and simultaneous collection of two images in the 480 nm and 535 nm channels was performed using a FRET module under a laser confocal microscope equipped with a 40-fold lens (Leica DM IRB Microsystems). The exposure time for both channels was set at 200 ms, with an interval of 20 s. Quantification of the specific signal involved subtracting the fluorescence intensity of nonspecific areas from the intensity of fluorescent cells expressing the sensor. Following a 5-minute baseline measurement, rat-FLSs were stimulated with Forskolin (10 μM) and IBMX (10 μM) or PGE_2_ (10 μM). The FRET ratio (CFP/YFP) was calculated for each individual FLSs.

### Experimental arthritis animal models and treatment

Three experimental arthritis animal models were used in this study. ***1) Mouse collagen-induced arthritis (CIA) model:*** The mouse CIA model was induced as previously described^27^. Briefly, male *Pde4d^+/+^* and *Pde4d^-/-^* mice (10-12-weeks-old) were intradermally immunized with 200 μg of chicken type Ⅱ collagen (20011, Chondrex, Redmond, WA, USA) emulsified in an equal volume of 10 mg/ml complete Freund's adjuvant (CFA) (7027, Chondrex, Redmond, WA, USA). On days 14 and 21, the mice were re-immunized by intradermally injecting chicken type Ⅱ collagen emulsified in incomplete Freund's adjuvant (7002, Chondrex, Redmond, WA, USA). ***2) Mouse collagen antibody-induced arthritis (CAIA) model:*** For the induction of the mouse CAIA model, male *Grk2^+/+^* and *Grk2^+/-^* mice (10-12-weeks-old) were intraperitoneally injected with 4 mg of anti-collagen-II 5-clone antibody cocktail (53100, Chondrex, Redmond, WA, USA) on day 0, followed by a subsequent intraperitoneal injection of 40 mg lipopolysaccharide on day 3, as previously described^26^. ***3) Rat CIA model and treatment:*** For the induction of the rat CIA model, male Sprague Dawley rats (8-10-weeks-old) were immunized by intradermal injection in the tail with 200 μg of chicken type Ⅱ collagen emulsified in an equal volume of 8 mg/ml CFA. On day 7, the rats were re-immunized with the chicken type Ⅱ collagen emulsion, as previously described^28^. After the onset of arthritis (approximately 14 days after the first immunization), CIA rats were randomly assigned to CIA + Vehicle, CIA + CP-25, and CIA + MTX groups. CP-25 (50 mg/kg/day), methotrexate (MTX) (0.5 mg/kg/every 3 days), or Vehicle were administered by gavage from day 14 to day 35 the after first immunization. CP-25 [C_29_H_32_O_13_S, molecular weight: 620], a white crystalline powder with purity>98%, was provided by the Chemistry Laboratory of the Institute of Clinical Pharmacology of Anhui Medical University (Hefei, China). MTX (2.5 mg per tablet) was purchased from XINYI Medical (Shanghai, China). The dosages of CP-25 and MTX were chosen based on our previous research^24^.

### Arthritis assessment

The swollen joint count, arthritis index, and global score were used to assess the severity of arthritis. Five phalanx joints and one ankle or wrist joint were included in the swollen joint count; therefore, the maximum count for each animal was 24. The arthritis index was scaled from 0 to 4 for each paw, resulting in a maximum score of 16. The arthritis index and the global scored as previously described^4^.

### Statistical analysis

All experiments were independently repeated at least three times. Statistical analyses were conducted using GraphPad Prism 9.4.1 software (GraphPad, CA, USA). Two-way analysis of variance and Student's t tests were employed to determine the differences between experimental groups. Data pertaining to histopathologic scores were presented by volume quartiles. Non-parametric Mann-Whitney U test was used to compare two groups, while Kruskal-Wallis test with Dunn's multiple comparison test correction was used to compare multiple groups.* p*-values < 0.05 were considered statistically significant.

## Results

### PDE4D exhibits abundant expression in the synovial tissues of both experimental arthritis animals and RA patients

**Figure 1:**
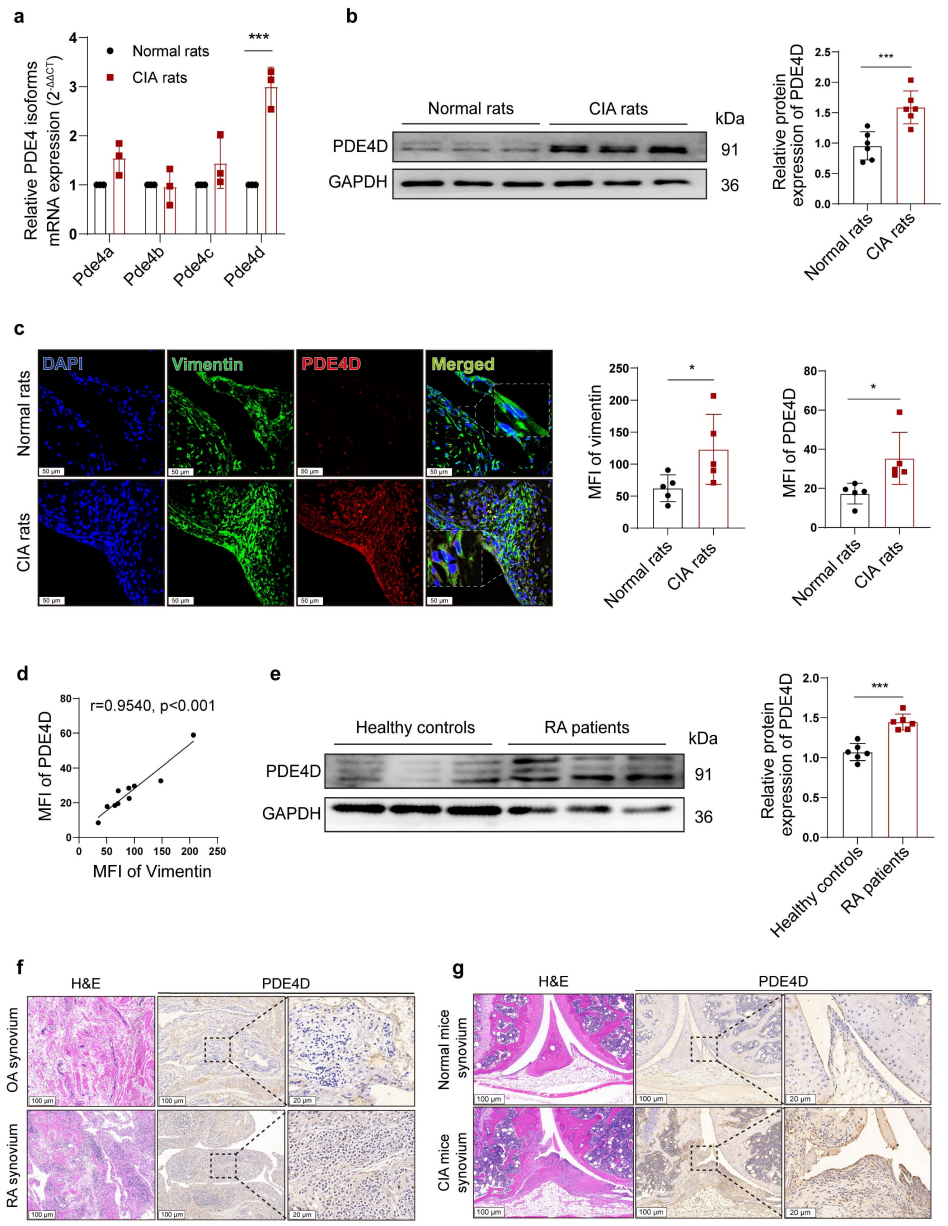
Expression of PDE4D in experimental arthritis models and patients with RA. (a) Transcript analysis of PDE4 isoforms in FLSs from synovial tissues of normal and CIA rats. n = 3. (b) Immunoblot analysis and quantification of PDE4D protein expression in FLSs from synovial tissues of normal and CIA rats. n = 6. (c) Representative IF (left) and quantification (right) of vimentin and PDE4D in synovial tissues of normal and CIA rats. n = 5. (d) Correlation analysis of vimentin MFI with PDE4D MFI in CIA mice synovial tissues. Two-tailed Spearman correlation test. (e) Immunoblot analysis and quantification of PDE4D protein expression in FLSs from synovial tissues of healthy controls and RA patients. n = 6. (f) Representative H&E staining images (rightmost) and IHC staining analysis of PDE4D protein expression in synovium of OA and RA patients. (g) Representative H&E staining images (rightmost) and IHC staining analysis of PDE4D protein expression in synovium of normal and CIA mice. Data are presented as mean ± SD from at least three independent experiments. ^*^p < 0.05 and ^***^p < 0.001.

PDE4 is encoded by four separate genes (*Pde4a-Pde4d*). To investigate which PDE4 subtype plays a crucial role in the abnormal proliferation of RA-FLSs, we successfully induced the rat CIA model (Fig. S1a-e). Notably, *Pde4d* mRNA, but not *Pde4a*, *Pde4b,* or *Pde4c*, exhibited a significant increase in CIA rat-FLSs compared with normal FLSs (Fig. 1a). Immunoblots confirmed a distinct up-regulation of PDE4D protein expression in CIA rat-FLSs compared with normal rats (Fig. 1b). Consistent with these findings, IF staining revealed heightened vimentin and PDE4D expression in CIA synovial tissues compared with normal tissues, especially in hyperproliferative FLSs (co-localized with vimentin-positive cells) (Fig. 1c). A strong positive correlation between the mean fluorescence intensity (MFI) of vimentin and PDE4D further supported these observations (Fig. 1d). Similar results were observed using protein lysates from FLSs of RA patients, where PDE4D protein was significantly overexpressed compared with healthy controls (Fig. 1e). IHC staining data also revealed increased PDE4D expression in the synovium of RA patients and CIA mice compared with that of OA patients and normal mice, respectively (Fig. 1f and g). Moreover, re-analysis of the microarray data (GSE181614) from human FLSs revealed marked upregulation of *Pde4d* mRNA in RA patients compared with trauma patients (Fig. S1f). These findings collectively demonstrate the highlighted PDE4D expression in the synovium of both experimental arthritis animals and RA patients, emphasizing its potential significance in RA pathogenesis.

### *Pde4d* deletion prevents arthritis development in CIA mice

**Figure 2:**
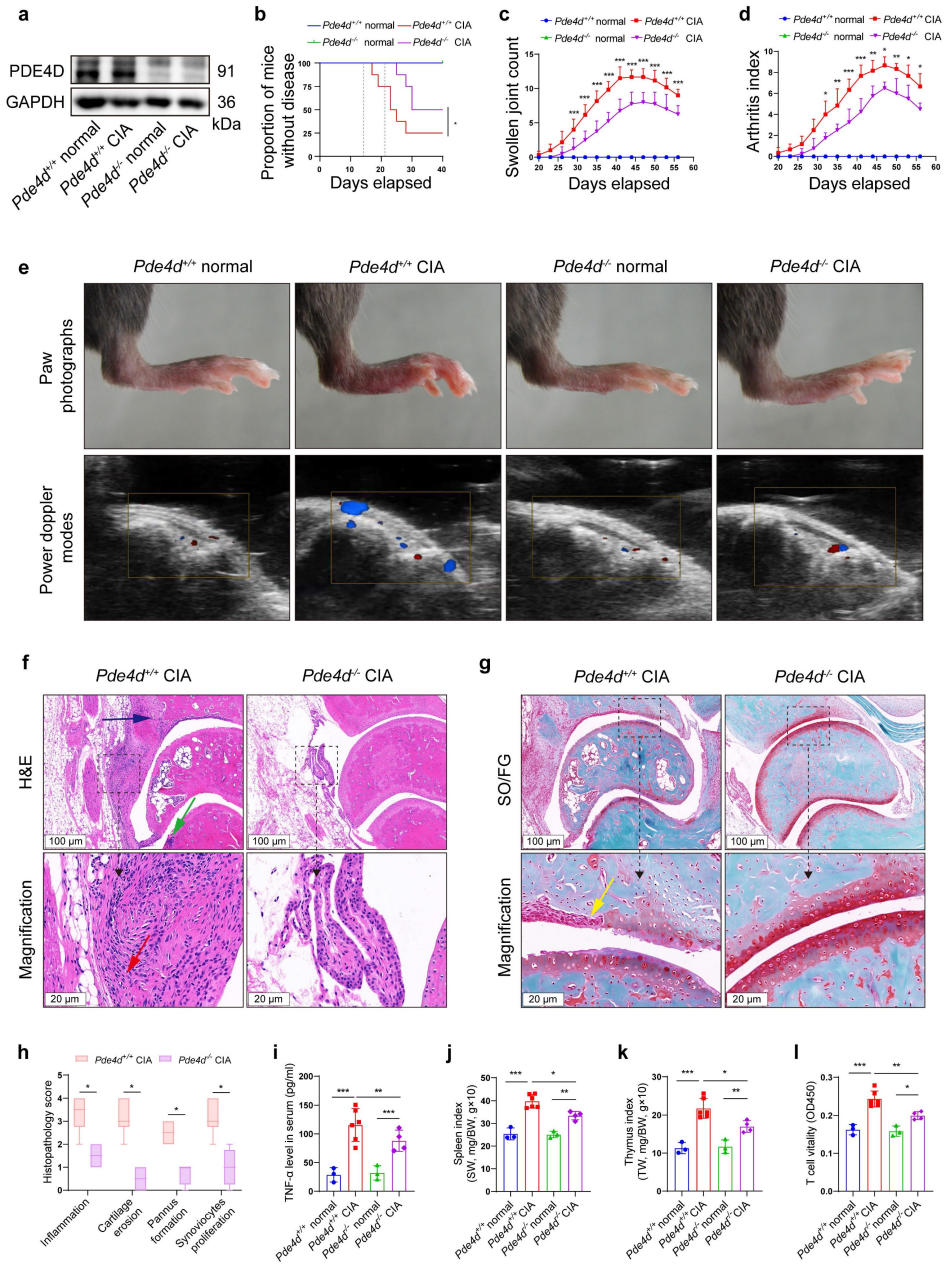
*Pde4d* deletion reduced arthritis severity in CIA mice. (a) Hind paws from normal and CIA mice were homogenized in cell lysis buffer, and PDE4D protein was detected by immunoblot, n = 4. (b) Arthritis incidence in CIA and normal mice. Logrank (Mantel-Cox) test. (c) Swollen joint count and (d) arthritis index of mice was recorded from the 20th day to the 56th day after the first immunization. n = 3-6. (e) Representative photographs of paws (upper) and power Doppler signals which reflecting vascularization and blood flow of synovium (lower) in different groups. (f) Representative images of H&E and (g) SO/FG staining of ankle joint sections from *Pde4d^+/+^* and *Pde4d^-/-^* CIA mice on the 56th day after the first immunization. Histopathologic changes include synovial hyperplasia (red arrowhead), pannus (blue arrowhead), infiltrating inflammatory cells (green arrowhead), and cartilage destruction (yellow arrowhead). (h) The histopathologic score inflammation, cartilage erosion, pannus formation, and synoviocytes proliferation of *Pde4d^+/+^* and *Pde4d^-/-^* CIA mice. n = 4-6. (i) The levels of TNF-α in mice serum were measured by ELISA. n = 3-6. (j) Spleen index (spleen weight (SW, mg)/bodyweight (BW, g) ×10) and (k) thymus index (thymus weight (TW, mg)/bodyweight (BW, g) ×10) in different groups. n = 3-6. (l) T cells vitality in different groups detected by CCK-8. n = 3-6. Data are presented as mean ± SD from at least three independent experiments. ^*^p < 0.05, ^**^p < 0.01 and ^***^p < 0.001.

To further elucidate the role of *Pde4d* in RA development, we induced the CIA model in both *Pde4d^+/+^* and *Pde4d^-/-^* mice. Deleting *Pde4d* (Fig. S2a-c and Fig. 2a) significantly reduced disease incidence, severity, swollen joint count, and arthritis index in *Pde4d^-/-^* CIA mice compared with *Pde4d^+/+^* CIA littermates (Fig. 2b-d). *Pde4d* knockout significantly ameliorated paw swelling and diminished power Doppler signals (reflecting vascularization and blood flow in the synovium) in CIA mice, while normal* Pde4d^+/+^* and *Pde4d^-/-^* mice (without immunization) showed no arthritis disorders (Fig. 2e). Moreover, histopathological analysis (HE and SO/FG staining) revealed milder arthritis symptoms in *Pde4d^-/-^* CIA mice, with reduced hyperplastic synovium, inflammatory cells, vascularization, cartilage destruction, and joint space narrowing compared with *Pde4d^+/+^* CIA mice (Fig. 2f-g). Additionally,* Pde4d* deletion lowered scores for inflammation, cartilage erosion, pannus formation, and synoviocytes proliferation compared with that of *Pde4d^+/+^* CIA mice (Fig. 2h). TNF-α levels were increased in CIA mice compared with normal mice. However, *Pde4d* knockout significantly reduced the increased TNF-α compared with *Pde4d* wild-type CIA mice (Fig. 2i). Interestingly, immunizing *Pde4d^+/+^* mice with chick type Ⅱ collagen showed enlarged spleens and thymus, whereas *Pde4d^-/-^* mice showed improvement (Fig. 2j-k). Furthermore, T-cell vitality was enhanced in *Pde4d^+/+^* CIA mice, whereas *Pde4d* ablation suppressed T-cell vitality (Fig. 2l). These results collectively demonstrate that *Pde4d* deletion effectively prevents arthritis development in CIA mice.

### Inhibiting PDE4D significantly ameliorates TNF-α-induced arthritic phenotypes of FLSs

**Figure 3:**
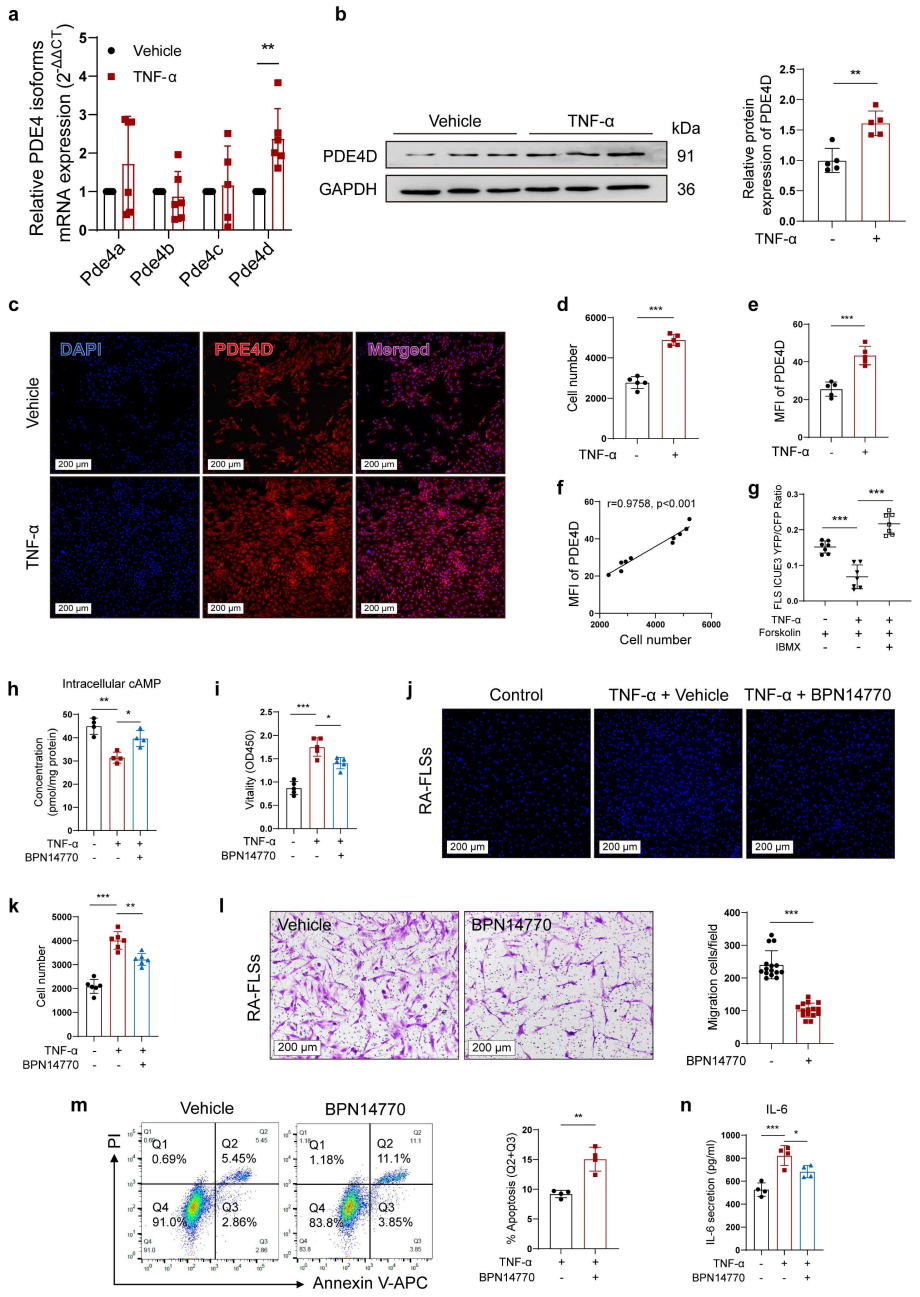
Inhibiting PDE4D significantly ameliorated TNF-α-induced arthritis phenotypes of FLSs. a-f, normal rat-FLSs were isolated and stimulated with TNF-α (20 ng/ml) or vehicle for 48 h. (a) Transcript analysis of PDE4 isoforms in FLSs. n = 6. (b) Immunoblot analysis and quantification of PDE4D protein expression in FLSs. n = 5. (c) Representative IF staining of DAPI and PDE4D, (d) quantification of cell number by HCCIS, and (e) MFI of PDE4D in FLSs. n = 5. (f) Correlation analysis of cell number with PDE4D MFI. Two-tailed Spearman correlation test. (g) cAMP contents in rat-FLSs after TNF-α, Forskolin or IBMX treatment were detected by FRET. n = 7. h-k and m-n, RA-FLSs were stimulated with TNF-α (20 ng/ml) and treated with BPN14770 for 48 h. (h) Intracellular cAMP contents in FLSs were detected by ELISA. n = 4. (i) Cellular vitality of FLSs detected by CCK-8. n = 5. (j) Representative images of cell number and (k) quantification of FLSs detected by HCCIS. n = 5. (l) Migration of RA-FLSs assessed and counted from six different patients and performed in triplicate after treatment with Vehicle and BPN14770. Five different fields were selected for cell counting. n = 6. (m) Representative flow cytometry plots and quantification of apoptotic FLSs. n = 4. (n) Concentrations of IL-6 in supernatant were measured by ELISA. n = 4. Data are presented as mean ± SD from at least three independent experiments. ^*^p < 0.05, ^**^p < 0.01 and ^***^p < 0.001.

RA involves uncontrolled FLSs proliferation induced by pro-inflammatory cytokines, particularly TNF-α^29^. To investigate the role of PDE4D in TNF-induced FLSs proliferation, normal rat-FLSs were isolated and treated with TNF-α (20 ng/ml)^4^ or vehicle. The expression of *Pde4d* mRNA was significantly increased, whereas the expression of *Pde4a*,* Pde4b*,* or Pde4c* remained unchanged (Fig. 3a), aligning with the mRNA expression pattern observed in rat CIA FLSs (Fig. 1a). Immunoblots confirmed an increase in PDE4D expression in rat-FLSs in response to TNF-α stimulation (Fig. 3b). In agreement with these results, TNF-α obviously promoted FLSs proliferation and upregulated PDE4D expression (Fig. 3c-e). Importantly, there was a strong positive correlation between cell number and the MFI of PDE4D (Fig. 3f). To investigate TNF-α's impact on intracellular cAMP, a FRET assay using the cAMP detection biosensor ICUE3 revealed reduced Forskolin-induced cAMP production by TNF-α, indicating suppressed intracellular cAMP levels (Fig. 3g). Subsequently, the TNF-α-mediated reduction in cAMP levels was reversed by IBMX (10 μM)^30^, a pan-PDEs inhibitor, suggesting that upregulated PDEs activity contributed to the TNF-α-impaired cAMP signaling in FLSs (Fig. 3g). Consistently, intracellular cAMP, reduced by TNF-α, was effectively restored by applying the PDE4D allosteric inhibitor BPN14770 (1 μM)^31^ (Fig. 3h). Taken together, these data confirm that TNF-α enhances PDE4D expression and activity, significantly inhibiting intracellular cAMP production in rat-FLSs.

Activated FLSs exhibit hyperproliferation and migration. Inhibiting PDE4D with BPN14770 (1 μM) significantly decreased rat-FLSs proliferation in response to TNF-α stimulation compared with the vehicle control (Fig. S3a-b). Meanwhile, BPN14770 treatment effectively inhibited TNF-α-evoked cell vitality (Fig. 3i), proliferation (Fig. 3j-k) and migration (Fig. 3l), while enhancing apoptosis of RA-FLSs (Fig. 3m). Furthermore, BPN14770 restrained TNF-α-induced IL-6 synthesis in RA-FLSs (Fig. 3n). These results collectively demonstrate that TNF-α-induced arthritic phenotypes in FLSs are mediated by upregulating PDE4D expression.

### GRK2 mediates overexpressed PDE4D in TNF-α-treated FLSs through the NF-κB p65 and ERK pathways

**Figure 4:**
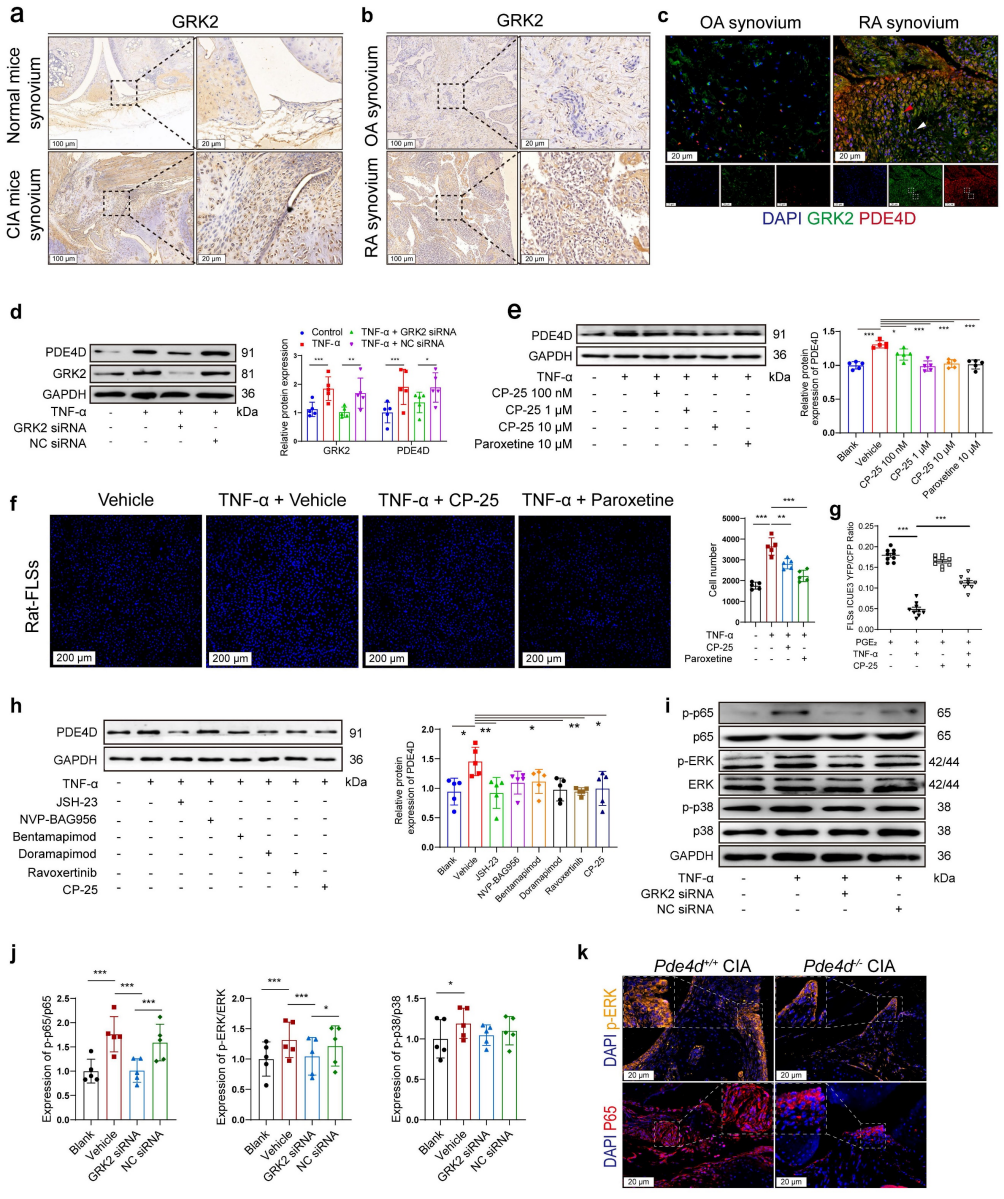
Overexpressed PDE4D in TNF-α-treated FLSs was mediated by GRK2. (a) Representative images of IHC staining of GRK2 from the synovium of normal and CIA mice. (b) Representative images of IHC staining of GRK2 from the synovium of OA and RA patients. (c) Representative double-staining IF of PDE4D and GRK2 from the synovium of OA and RA patients. (d) Immunoblot analysis and quantification of PDE4D and GRK2 protein expression in rat-FLSs after transfected with GRK2 siRNA and NC siRNA. n = 5. (e) Immunoblot analysis and quantification of PDE4D protein expression in rat-FLSs after treated with GRK2 inhibitors. n = 5. (f) Representative images of cell number (left) and quantification (right) of rat-FLSs after treated with GRK2 inhibitors detected by HCCIS. n = 5. (g) cAMP contents in rat-FLSs after treated with PGE_2_, TNF-α and CP-25 were detected by FRET. n = 9. (h) Immunoblot analysis and quantification of PDE4D protein expression in rat-FLSs after treated with different pathway inhibitors. n = 5. (i) Immunoblot analysis and quantification (j) of p-p65, p65, p-ERK, ERK, p-p38, and p38 protein expression in rat-FLSs after transfected with GRK2 siRNA and NC siRNA. n = 5. (k) Representative p-ERK and P65 IF staining in the synovium of CIA mice. Data are presented as mean ± SD from at least three independent experiments. ^*^p < 0.05, ^**^p < 0.01 and ^***^p < 0.001.

We have previously reported that GRK2, abundantly expressed in TNF-α-treated FLSs, is crucial for FLSs proliferation^4^. IHC staining of synovium from CIA mice and RA patients showed increased GRK2 expression compared with normal mice and OA patients (Fig. 4a-b), respectively. Notably, compared with OA patients, GRK2 and PDE4D expression were obviously elevated in the synovium of RA patients, with elevated GRK2 levels correlating with increased PDE4D expression and vice versa (as indicated by the red and white arrows) (Fig. 4c). The consistent expression patterns of GRK2 and PDE4D suggested a potential regulatory role of GRK2 in PDE4D expression. As validated, TNF-α (20 ng/ml) stimulation increased GRK2 expression in rat-FLSs (Fig. S4a-b). Importantly, TNF-α-induced PDE4D expression was eliminated by GRK2 deficiency (Fig. 4d and Fig. S4c-d). Moreover, inhibiting GRK2 with paroxetine^4^ or the novel GRK2 inhibitor CP-25, an esterification modification of paeoniflorin, significantly prevented TNF-α-induced PDE4D expression (Fig. 4e). Conversely, GRK2 overexpression (OE) directly upregulated PDE4D protein expression in rat-FLSs (Fig. S4e-g). These results indicate that TNF-α-mediated upregulation of PDE4D may dependent on GRK2.

Consistent with the reduced PDE4D expression observed with GRK2 inhibitors, CP-25 or paroxetine attenuated TNF-α-induced rat-FLSs proliferation (Fig. 4f). Prostaglandin E_2_ (PGE_2_), a ligand of the prostaglandin receptor, was used to increase intracellular cAMP content^4^. TNF-α (20 ng/ml) pre-treatment robustly reduced PGE_2_-stimulated cAMP concentration, potentially due to TNF-α-induced upregulation of PDE4D (Fig. 4g). CP-25 (1 μM) significantly reversed the TNF-α-mediated reduction of cAMP production in response to PGE_2_, whereas CP-25 alone had a limited effect on PGE_2_-induced cAMP synthesis (Fig. 4g). These findings collectively indicate that TNF-α-induced GRK2 promotes PDE4D expression, leading to a reduction in intracellular cAMP concentration and the hyperproliferation of FLSs.

While the connection between GRK2 and PDE4D in FLSs is established, the exact mechanism remains unclear. GRK2 influences downstream molecules through various pathways, including MAPK, PI3K, ERK and NF-κB^22^, ^32^. To explore the pathway by which GRK2 regulates PDE4D, TNF-stimulated FLSs were treated with inhibitors of different pathways, including the NF-κB inhibitor (JSH-23, 10 μM)^33^, PI3K inhibitor (NVP-BAG956, 10 μM)^34^, JNK inhibitor (Bentamapimod, 7.5 μM)^35^, p38 inhibitor (Doramapimod, 10 μM)^36^, ERK inhibitor (Ravoxertinib, 5 μM)^37^ and CP-25 (1 μM)^24^. We found that NF-κB, p38, and ERK inhibitors, as well as CP-25, significantly suppressed TNF-α-induced PDE4D upregulation, while PI3K and JNK inhibitors had limited effects (Fig. 4h). Knocking down GRK2 with siRNA inhibited p65 and ERK phosphorylation but had a limited effect on p38 phosphorylation in TNF-α-stimulated rat-FLSs (Fig. 4i-j). Moreover, there was no difference in GRK2 expression between *Pde4d^+/+^* and *Pde4d^-/-^* CIA mice (Fig. S4h-i). The expression of p65, particularly in the nucleus, and p-ERK were notably reduced in *Pde4d^-/-^* CIA mice compared with *Pde4d^+/+^* CIA mice (Fig. 4k and Fig. S4j-k). These results demonstrate that TNF-α-induced upregulation of PDE4D in rat-FLSs is, at least partially, dependent on GRK2-mediated activation of the p65 and ERK pathways.

### *Grk2*-deficient mice decrease PDE4D and relieve arthritic symptoms in CAIA

**Figure 5:**
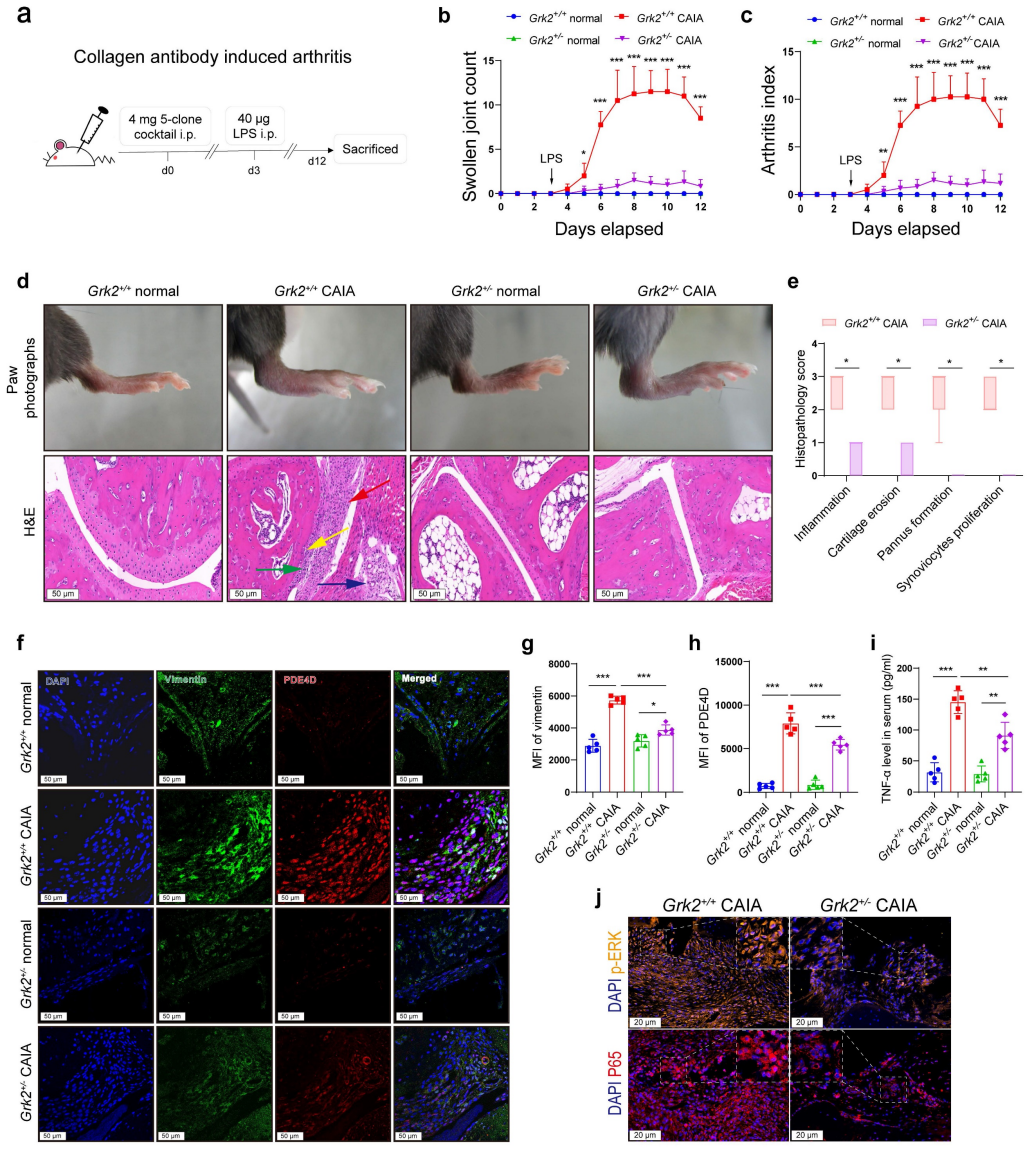
*Grk2*-deficient mice relieved symptoms in CAIA. (a) Male *Grk2^+/+^* and *Grk2^+/-^* mice were used to establish a CAIA mouse model as indicated. (b) Swollen joint count and (c) arthritis index of mice were recorded from the 0th day to the 12th day after intraperitoneal injection of mouse monoclonal anti-collagen-II 5-clone antibody cocktail. n = 5. (d) Representative photographs of paws (upper) and H&E staining (lower) of the ankle joint sections from different groups. Histopathologic changes include synovial hyperplasia (red arrowhead), pannus (blue arrowhead), infiltrating inflammatory cells (green arrowhead), and cartilage destruction (yellow arrowhead). (e) Histopathological score of inflammation, cartilage erosion, pannus formation, and synoviocytes proliferation of *Grk2^+/+^* and *Grk2^+/-^* CAIA mice. n = 5. (f) Representative double-staining IF of PDE4D and GRK2 from the synovial tissues of normal and CAIA mice. (g) Quantification of vimentin and (h) PDE4D MFI in the synovial tissues of normal and CAIA mice. n = 5. (i) The levels of TNF-α in mice serum were measured by ELISA. n = 5. (j) Representative p-ERK and P65 IF staining in the synovium of CAIA mice. Data are presented as mean ± SD from at least five independent experiments. ^*^p < 0.05, ^**^p < 0.01 and ^***^p < 0.001.

To elucidate the specific role of GRK2 in PDE4D expression and experimental arthritis* in vivo*, *Grk2*-deficient and *Grk2^+/+^* littermate mice were used as experimental animals to induce the CAIA model (Fig. 5a), another well-established experimental arthritis model^26^. As global *Grk2* knockout (*Grk2^-/-^*) mice are embryonically lethal^38^, *Grk2* heterozygous knockout mice (*Grk2^+/-^*) were utilized. Immunoblots revealed that *Grk2^+/-^* mice exhibited a significant reduction in GRK2 protein expression (Fig. S5a-b), which was consistent with previous report^23^, ^39^. Following induction of the CAIA model, *Grk2^+/+^* CAIA mice exhibited severe joint inflammation and paw swelling, peaking around days 8-11 (Fig. 5b-c). In contrast, *Grk2^+/-^* CAIA mice showed a significant reduction in swollen joint counts (Fig. 5b) and arthritis index (Fig. 5c).

Moreover, *Grk2^+/+^* CAIA mice, compared with *Grk2^+/-^* CAIA mice, exhibited more severe symptoms, including swollen ankles and thick paws (Fig. 5d), along with synovium hyperplasia, inflammatory cells infiltration, extensive pannus formation, and cartilage destruction (Fig. 5d). Normal* Grk2^+/+^* and *Grk2^+/-^* mice (without immunization) showed no signs of arthritis disorders (Fig. 5b-d). In contrast, *Grk2^+/-^* CAIA mice displayed synovial tissues with only one or two layers of synoviocytes, devoid of infiltrated inflammatory cells or damaged cartilage (Fig. 5d). Additionally, *Grk2^+/-^* CAIA mice had significantly lower histopathology scores compared with *Grk2^+/+^* CAIA mice (Fig. 5e). Furthermore, IF staining demonstrated that vimentin and PDE4D staining were strongly enhanced and highly co-localized in synovium sections from *Grk2^+/+^* CAIA mice (Fig. 5f-h). In contrast, *Grk2^+/-^* CAIA mice exhibited reduced vimentin and PDE4D staining (Fig. 5f-h). Compared with normal mice, TNF-α levels were increased in CAIA mice. However, the increased TNF-α levels were significantly reduced in *Grk2^+/-^* CAIA mice when compared with *Grk2^+/+^* CAIA mice (Fig. 5i). Furthermore, the expression of p65, especially in the nucleus, and p-ERK were significantly reduced in *Grk2^+/-^* CAIA mice compared with *Grk2^+/+^* CAIA mice (Fig. 5j and Fig. S5c-d). Collectively, these results demonstrate that *Grk2* deficiency reduces PDE4D expression and effectively ameliorates the severity and progression of experimental arthritis.

### Pharmacological inhibition of GRK2 attenuates arthritic symptoms of CIA rats by inhibiting PDE4D expression

**Figure 6:**
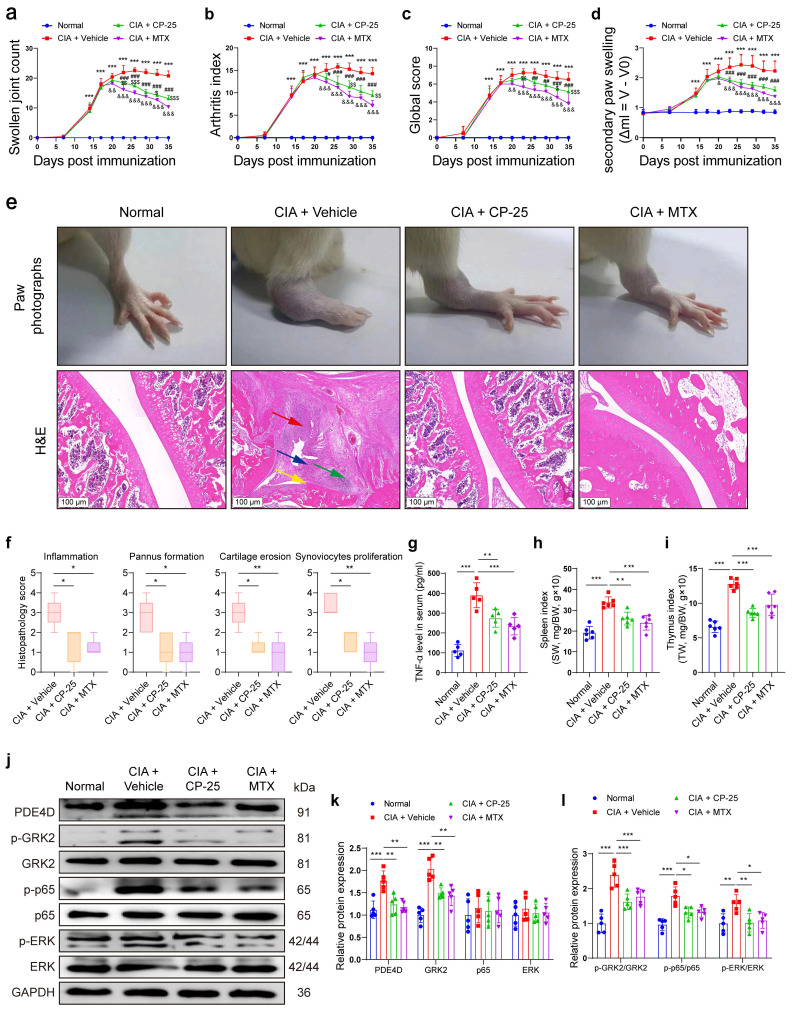
Pharmacological inhibition of GRK2 attenuated symptoms of CIA rats. Rats immunized with CII/CFA were randomly assigned to CIA + Vehicle, CIA + CP-25 and CIA + MTX. (a) Swollen joint count, (b) arthritis index, (c) global score, and (d) secondary paw swelling of mice were recorded from the 0th day to the 35th day after the first immunization. ^***^p < 0.001 versus normal group; *^#^p* < 0.05, *^##^p* < 0.01 and *^###^p* < 0.001 versus CIA model group; *^&^p* < 0.05, *^&&^p* < 0.01 and *^&&&^p* < 0.001 versus CIA model group; *^$^p* < 0.05, *^$$^p* < 0.01 and *^$$$^p* < 0.001 versus MTX group. n = 6. (e) Representative photographs of paws (upper) and H&E staining (lower) of the ankle joint sections from different groups. Histopathologic changes include synovial hyperplasia (red arrowhead), pannus (blue arrowhead), infiltrating inflammatory cells (green arrowhead), and cartilage destruction (yellow arrowhead). (f) Histopathological score of inflammation, pannus formation, cartilage erosion, and synoviocytes proliferation in different groups. n = 5. (g) The levels of TNF-α in rat serum were measured by ELISA. n = 5. (h) Spleen index (spleen weight (SW, mg)/bodyweight (BW, g) ×10) and (i) thymus index (thymus weight (TW, mg)/bodyweight (BW, g) ×10) in different groups. n = 6. (j) Immunoblot analysis and quantification (k and l) of PDE4D, p-GRK2, GRK2, p-p65, p65, p-ERK, and ERK protein expression from synovial tissues of different groups. n = 5. Data are presented as mean ± SD from at least five independent experiments. ^*^p < 0.05, ^**^p < 0.01 and ^***^p < 0.001.

To determine the regulatory effect of GRK2 on PDE4D and the therapeutic effect of the GRK2 inhibitor CP-25 *in vivo*, we successfully induced rat CIA model and administered with Vehicle, CP-25 or MTX. Rats subjected to CIA induction developed severe arthritic symptoms, while CP-25 or MTX significantly alleviated these symptoms, reducing swollen joint count, arthritis index, global score and joint swelling (Fig. 6a-d). Visually, collagen-induced redness and swelling in ankles were observed in CIA + Vehicle rats, while these phenotypes were relieved by CP-25 or MTX treatment (Fig. 6e). Additionally, histopathological analysis demonstrated that CP-25 and MTX effectively mitigated synovial hyperplasia, pannus formation, inflammatory cell infiltration, and tissue damage (Fig. 6e-f). Notably, treatment with CP-25 or MTX significantly reduced the increased TNF-α levels (Fig. 6g), and moderated the enlarged spleens and thymus in CIA model rats (Fig. 6h-i). Protein analysis of synovial tissues showed elevated PDE4D, GRK2, p-p65, and p-ERK in CIA + Vehicle rats compared with normal rats, which were significantly suppressed by CP-25, aligning with *in vitro* findings (Fig. 6j-l). MTX also exhibited a slight reduction in these markers, likely due to its anti-inflammatory and immunomodulatory effects (Fig. 6j-l). In summary, these results emphasize that GRK2 inhibition by CP-25 effectively alleviates CIA symptoms by suppressing PDE4D through the p65 and ERK pathways.

## Discussion

In the healthy synovium, FLSs play a role in producing synovial fluid, offering lubrication and nutrients to the cartilage surface. However, during the progression of RA, FLSs undergo transformation into hyperplastic and aggressive phenotypes, thereby contributing to persistent inflammation and advancing disability^40^. Consequently, targeting of hyperproliferative FLSs emerges as a promising therapeutic strategy to mitigate the progression of RA.

Intracellular cAMP crucially regulates FLSs proliferation and is regulated by both PDEs and GRK2-mediated GPCRs desensitization^4^. Previous studies have demonstrated that cAMP inhibits cell proliferation by suppressing the ERK pathway^41^. In activated FLSs, however, the cAMP concentration is decreased^5^, which is attributed to GRK2-mediated internalization and desensitization of prostaglandin E receptor 4 (EP4), thereby fostering FLSs proliferation, particularly as RA progresses to advanced stages^4^. However, these previous studies have not focused on the underlying causes of reduced cAMP levels in FLSs or the possible regulatory mechanisms involved. In addition, PDE4D has been reported to be upregulated in RA synovium and TNF-α treated FLSs^6^. Yet, the reasons for the increased expression of PDE4D in the synovium of RA patients and its specific role in FLSs remain unexplored. In the present study, we demonstrate that the reduced cAMP levels in FLSs are caused by the elevated PDE4D and that PDE4D is involved in the abnormal proliferation of FLSs, leading to the worsening of arthritis. Our study reveals abundant PDE4D expression in both synovial tissues from experimental arthritis animals and RA patients. Notably, TNF-α stimulation amplifies PDE4D expression, correlating with enhanced FLSs proliferation. However, intervention with BPN14770 effectively ameliorated the arthritic phenotypes of FLSs. To further illustrate the pathogenic role of PDE4D in RA, we established a global *Pde4d^-/-^* mouse line and induced a CIA model. Our results demonstrated that normal *Pde4d^-/-^* mice didn't exhibit developmental defects or arthritis disorders, which may be explained by functional redundancy among PDEs members^42^. Strikingly, global *Pde4d* deletion significantly alleviated the severity and progression of arthritis induced by collagen. In this experiment, *Pde4d^-/-^* CIA mice demonstrated reduced disease incidence and severity, exemplified by a diminished arthritis index. More importantly, *Pde4d^-/-^* CIA mice exhibited remarkable mitigation in pathological changes, including synovial hyperplasia, inflammatory cell infiltration, pannus formation, as well as cartilage destruction. These results provided compelling evidence that *Pde4d* deletion profoundly mitigates arthritis severity and prevents cartilage deterioration in CIA mice, emphasizing potential therapeutic avenues for RA.

GRK2 emerges as a promising target to inhibit the hyperactivation of FLSs in RA^24^. Studies have indicated that elevated GRK2 expression induces GPCR desensitization, suppressing Gαs-cAMP signaling and decreasing cAMP content^4^, ^43^, ^44^. Conversely, cAMP content was increased in *Grk2* conditional knockout chondrocytes^44^. Our previous studies confirmed diminished cAMP content in FLSs of CIA rats following treatment with TNF-α^4^, ^5^. According to previous reports, there is a certain relationship between GRK2 and cAMP. However, whether GRK2 affects cAMP levels by regulating PDE4D and its possible mechanism have not been reported. Therefore, in the present study, we hypothesized that TNF-α-mediated cAMP reduction is intricately linked with GRK2. In this study, we confirmed GRK2 overexpression in the synovium of RA patients and arthritis animal models, demonstrating its co-localization with PDE4D in RA synovium and its regulation of PDE4D expression in FLSs. Mechanistically, GRK2 orchestrated PDED4 expression through the NF-κB p65 and ERK pathways. Crucially, utilizing* Grk2*-deficient mice, we showed that endogenous *Grk2* knockdown significantly decreased PDE4D expression, alleviating arthritis symptoms in the CAIA model. Furthermore, pharmacological inhibition of GRK2 by CP-25 substantially suppressed PDE4D expression and impeded arthritis development in the CIA model. These results not only confirm the pivotal and causal role of PDE4D in RA-FLSs but also provide a novel molecular insight into how GRK2 regulates PDE4D expression. These findings further establish a proof of concept for targeting GRK2 to alleviate arthritis *in vivo*.

**Figure 7:**
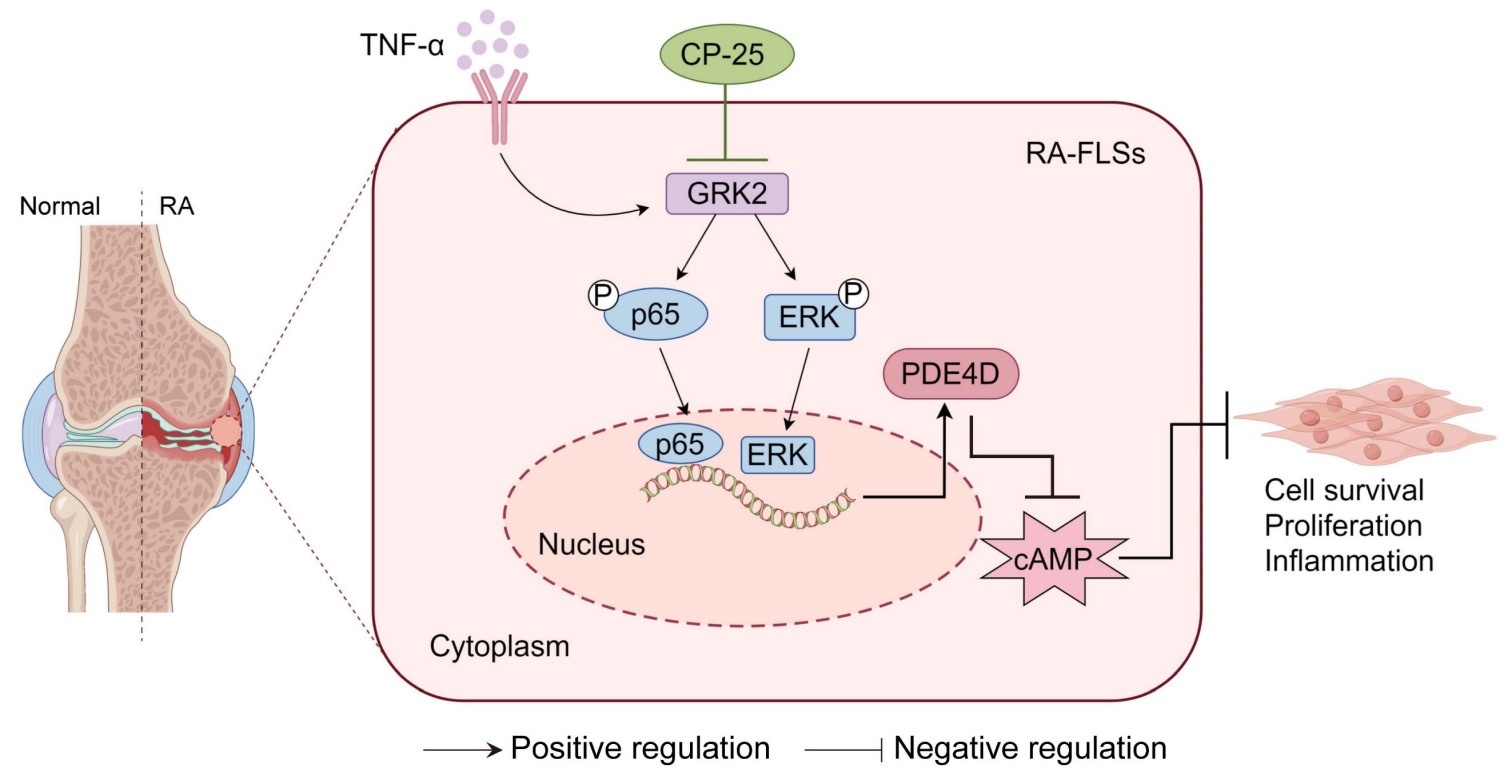
A schematic illustration summarizing the regulation and function of the GRK2-PDE4D axis in RA-FLSs. TNF-α-stimulated GRK2 increases PDE4D expression via the p65 and ERK pathways, resulting in a decrease in cAMP concentration and the promotion of FLSs proliferation. CP-25 inhibits the GRK2-PDE4D axis, thereby suppressing hyperproliferation of FLSs and alleviating experimental arthritis. The figure was created using Figdraw.

Briefly, the upregulated PDE4D in FLSs is dependent on GRK2 and its downstream p65 and ERK pathways. Global deletion of *Pde4d* reduces the arthritis incidence and severity in CIA mice. Both genetic deficiency and pharmacological inhibition of GRK2 have remarkably reduced PDE4D expression, consequently ameliorating the severity and progression of arthritis in animal models (Fig. 7).

## Conclusion

Collectively, our study unveils an essential, previously unrecognized role of PDE4D in RA pathogenesis, along with the regulation of PDE4D expression in FLSs by GRK2. These findings propose the GRK2-PDE4D axis as a potent and novel therapeutic target for RA.

### Limitations

Regarding the series of experiments conducted in the present investigation, several limitations cannot be ignored. Firstly, it would be more convincing to induce an experimental arthritis model in FLSs-specific *Pde4d* or *Grk2* deletion mice. Secondly, the sample of RA patients and healthy donors included in the present study is relatively limited. Finally, the detailed molecular mechanism of GRK2-regulated PDE4D expression needs to be further elucidation. In future investigations, we aim to establish mice with specific knockout of *Pde4d* or *Grk2* in FLSs and induce experimental arthritis models. We will also continue to recruit more RA patients and healthy donors. Furthermore, we will employ additional molecular biological approaches to elucidate the detailed molecular mechanisms by which GRK2 regulates PDE4D expression, including how GRK2 interacts with these pathways and how these pathways affect PDE4D expression.

## Supplementary Material

Supplementary figures and tables.

## Abbreviations

CAIA: collagen antibody-induced arthritis
cAMP: cyclic adenosine monophosphate
CFA: complete Freund's adjuvant
CIA: collagen-induced arthritis
CII: type II collagen
ELISA: enzyme-linked immunosorbent assay
EP4: prostaglandin E receptor 4
ERK: extracellular signal-regulated kinase
FLSs: fibroblast-like synoviocytes
FRET: fluorescence resonance energy transfer
GPCRs: G protein-coupled receptors
GRK2: G protein coupled receptor kinase 2
H&E: hematoxylin and eosin
HCCIS: high-content cell imaging system
IF: immunofluorescence
IFA: incomplete Freund's adjuvant
IHC: immunohistochemical
IL-6: interleukin-6
JNK: c-Jun N-terminal kinase
MAPK: mitogen-activated protein kinase
MFI: mean fluorescence intensity
MTX: methotrexate
NF-κB: nuclear factor kappa-B
OA: osteoarthritis
OE: overexpression
PDE4D: phosphodiesterase 4D
PGE2: prostaglandin E2
PI3K: phosphatidylinositol 3-kinase
qPCR: quantitative PCR
RA: rheumatoid arthritis
SD: standard deviation
SO/FG: safranin O-fast green
TNF-α: tumor necrosis factor-α

## References

[1] Van der Woude D, van der Helm-van Mil AHM (2018). Update on the epidemiology, risk factors, and disease outcomes of rheumatoid arthritis. Best Pract Res Clin Rheumatol.

[2] Raker VK, Becker C, Steinbrink K (2016). The cAMP Pathway as Therapeutic Target in Autoimmune and Inflammatory Diseases. Front Immunol.

[3] He Y, Huang Y, Mai C, Pan H, Luo HB, Liu L (2020). The immunomodulatory role of PDEs inhibitors in immune cells: therapeutic implication in rheumatoid arthritis. Pharmacol Res.

[4] Tai Y, Huang B, Guo PP, Wang Z, Zhou ZW, Wang MM (2022). TNF-alpha impairs EP4 signaling through the association of TRAF2-GRK2 in primary fibroblast-like synoviocytes. Acta Pharmacol Sin.

[5] Chang Y, Wei W, Zhang L, Xu HM (2009). Effects and mechanisms of total glucosides of paeony on synoviocytes activities in rat collagen-induced arthritis. J Ethnopharmacol.

[6] De Groof A, Ducreux J, Humby F, Nzeusseu Toukap A, Badot V, Pitzalis C (2016). Higher expression of TNFalpha-induced genes in the synovium of patients with early rheumatoid arthritis correlates with disease activity, and predicts absence of response to first line therapy. Arthritis Res Ther.

[7] McCann FE, Palfreeman AC, Andrews M, Perocheau DP, Inglis JJ, Schafer P (2010). Apremilast, a novel PDE4 inhibitor, inhibits spontaneous production of tumour necrosis factor-alpha from human rheumatoid synovial cells and ameliorates experimental arthritis. Arthritis Res Ther.

[8] Chen W, Wang J, Xu Z, Huang F, Qian W, Ma J (2018). Apremilast Ameliorates Experimental Arthritis via Suppression of Th1 and Th17 Cells and Enhancement of CD4(+)Foxp3(+) Regulatory T Cells Differentiation. Front Immunol.

[9] Fang R, Cui Q, Sun J, Duan X, Ma X, Wang W (2015). PDK1/Akt/PDE4D axis identified as a target for asthma remedy synergistic with beta2 AR agonists by a natural agent arctigenin. Allergy.

[10] Wade SM, Trenkmann M, McGarry T, Canavan M, Marzaioli V, Wade SC (2019). Altered expression of microRNA-23a in psoriatic arthritis modulates synovial fibroblast pro-inflammatory mechanisms via phosphodiesterase 4B. J Autoimmun.

[11] Tenor H, Hedbom E, Hauselmann HJ, Schudt C, Hatzelmann A (2002). Phosphodiesterase isoenzyme families in human osteoarthritis chondrocytes-functional importance of phosphodiesterase 4. Br J Pharmacol.

[12] Contreras S, Milara J, Morcillo E, Cortijo J (2017). Selective Inhibition of Phosphodiesterases 4A, B, C and D Isoforms in Chronic Respiratory Diseases: Current and Future Evidences. Curr Pharm Des.

[13] Silverberg JI, French LE, Warren RB, Strober B, Kjoller K, Sommer MOA (2023). Pharmacology of orismilast, a potent and selective PDE4 inhibitor. J Eur Acad Dermatol Venereol.

[14] Li H, Zhang X, Xiang C, Feng C, Fan C, Liu M (2021). Identification of phosphodiesterase-4 as the therapeutic target of arctigenin in alleviating psoriatic skin inflammation. J Adv Res.

[15] Li G, He D, Qian X, Liu Y, Ou Y, Li M (2024). Development of selective heterocyclic PDE4 inhibitors for treatment of psoriasis. Eur J Med Chem.

[16] Deng YM, Xie QM, Tang HF, Sun JG, Deng JF, Chen JQ (2006). Effects of ciclamilast, a new PDE 4 PDE4 inhibitor, on airway hyperresponsiveness, PDE4D expression and airway inflammation in a murine model of asthma. Eur J Pharmacol.

[17] Berry-Kravis EM, Harnett MD, Reines SA, Reese MA, Ethridge LE, Outterson AH (2021). Inhibition of phosphodiesterase-4D in adults with fragile X syndrome: a randomized, placebo-controlled, phase 2 clinical trial. Nat Med.

[18] Gurney ME, Nugent RA, Mo X, Sindac JA, Hagen TJ, Fox D 3rd (2019). Design and Synthesis of Selective Phosphodiesterase 4D (PDE4D) Allosteric Inhibitors for the Treatment of Fragile X Syndrome and Other Brain Disorders. J Med Chem.

[19] Zhang Y, Zhang J, Wang J, Chen H, Ouyang L, Wang Y (2022). Targeting GRK2 and GRK5 for treating chronic degenerative diseases: Advances and future perspectives. Eur J Med Chem.

[20] Wang DD, Jiang MY, Wang W, Zhou WJ, Zhang YW, Yang M (2020). Paeoniflorin-6'-O-benzene sulfonate down-regulates CXCR4-Gbetagamma-PI3K/AKT mediated migration in fibroblast-like synoviocytes of rheumatoid arthritis by inhibiting GRK2 translocation. Biochem Biophys Res Commun.

[21] Zhang BJ, Wang YY, Jia CY, Li SS, Wang XW, Xu Y (2022). Paeoniflorin-6'-o-benzene sulfonate ameliorates the progression of adjuvant-induced arthritis by inhibiting the interaction between Ahr and GRK2 of fibroblast-like synoviocytes. Int Immunopharmacol.

[22] Penela P, Murga C, Ribas C, Lafarga V, Mayor F Jr (2010). The complex G protein-coupled receptor kinase 2 (GRK2) interactome unveils new physiopathological targets. Br J Pharmacol.

[23] Han D, Jiang C, Xu H, Chu R, Zhang R, Fang R (2024). Inhibition of GRK2 ameliorates the pristane-induced mouse SLE model by suppressing plasma cells differentiation. Int Immunopharmacol.

[24] Han C, Li Y, Zhang Y, Wang Y, Cui D, Luo T (2021). Targeted inhibition of GRK2 kinase domain by CP-25 to reverse fibroblast-like synoviocytes dysfunction and improve collagen-induced arthritis in rats. Acta Pharm Sin B.

[25] Huang B, Wang QT, Song SS, Wu YJ, Ma YK, Zhang LL (2012). Combined use of etanercept and MTX restores CD4(+)/CD8(+) ratio and Tregs in spleen and thymus in collagen-induced arthritis. Inflamm Res.

[26] Tu J, Han D, Fang Y, Jiang H, Tan X, Xu Z (2022). MicroRNA-10b promotes arthritis development by disrupting CD4(+) T cell subtypes. Mol Ther Nucleic Acids.

[27] Yin Y, Yang X, Wu S, Ding X, Zhu H, Long X (2022). Jmjd1c demethylates STAT3 to restrain plasma cell differentiation and rheumatoid arthritis. Nat Immunol.

[28] Han L, Zhang XZ, Wang C, Tang XY, Zhu Y, Cai XY (2020). IgD-Fc-Ig fusion protein, a new biological agent, inhibits T cell function in CIA rats by inhibiting IgD-IgDR-Lck-NF-kappaB signaling pathways. Acta Pharmacol Sin.

[29] Tu J, Hong W, Zhang P, Wang X, Korner H, Wei W (2018). Ontology and Function of Fibroblast-Like and Macrophage-Like Synoviocytes: How Do They Talk to Each Other and Can They Be Targeted for Rheumatoid Arthritis Therapy?. Front Immunol.

[30] Wu H, Lee J, Vincent LG, Wang Q, Gu M, Lan F (2015). Epigenetic Regulation of Phosphodiesterases 2A and 3A Underlies Compromised beta-Adrenergic Signaling in an iPSC Model of Dilated Cardiomyopathy. Cell Stem Cell.

[31] Zhang C, Xu Y, Chowdhary A, Fox D 3rd, Gurney ME, Zhang HT (2018). Memory enhancing effects of BPN14770, an allosteric inhibitor of phosphodiesterase-4D, in wild-type and humanized mice. Neuropsychopharmacology.

[32] Han CC, Liu Q, Zhang Y, Li YF, Cui DQ, Luo TT (2020). CP-25 inhibits PGE2-induced angiogenesis by down-regulating EP4/AC/cAMP/PKA-mediated GRK2 translocation. Clin Sci (Lond).

[33] Shin HM, Kim MH, Kim BH, Jung SH, Kim YS, Park HJ (2004). Inhibitory action of novel aromatic diamine compound on lipopolysaccharide-induced nuclear translocation of NF-kappaB without affecting IkappaB degradation. FEBS Lett.

[34] Bressanin D, Evangelisti C, Ricci F, Tabellini G, Chiarini F, Tazzari PL (2012). Harnessing the PI3K/Akt/mTOR pathway in T-cell acute lymphoblastic leukemia: eliminating activity by targeting at different levels. Oncotarget.

[35] Li Z, Sun C, Tao S, Osunkoya AO, Arnold RS, Petros JA (2020). The JNK inhibitor AS602801 Synergizes with Enzalutamide to Kill Prostate Cancer Cells In Vitro and In Vivo and Inhibit Androgen Receptor Expression. Transl Oncol.

[36] Kuma Y, Sabio G, Bain J, Shpiro N, Marquez R, Cuenda A (2005). BIRB796 inhibits all p38 MAPK isoforms in vitro and in vivo. J Biol Chem.

[37] Zhou J, Que Y, Pan L, Li X, Zhu C, Jin L (2022). Supervillin Contributes to LPS-induced Inflammatory Response in THP-1 Cell-derived Macrophages. Inflammation.

[38] Jaber M, Koch WJ, Rockman H, Smith B, Bond RA, Sulik KK (1996). Essential role of beta-adrenergic receptor kinase 1 in cardiac development and function. Proc Natl Acad Sci U S A.

[39] Eijkelkamp N, Heijnen CJ, Willemen HL, Deumens R, Joosten EA, Kleibeuker W (2010). GRK2: a novel cell-specific regulator of severity and duration of inflammatory pain. J Neurosci.

[40] You S, Koh JH, Leng L, Kim WU, Bucala R (2018). The Tumor-Like Phenotype of Rheumatoid Synovium: Molecular Profiling and Prospects for Precision Medicine. Arthritis Rheumatol.

[41] Doherty GA, Byrne SM, Molloy ES, Malhotra V, Austin SC, Kay EW (2009). Proneoplastic effects of PGE mediated by EP4 receptor in colorectal cancer. Bmc Cancer.

[42] He YF, Huang YD, Mai CT, Pan HD, Luo HB, Liu L (2020). The immunomodulatory role of PDEs inhibitors in immune cells: therapeutic implication in rheumatoid arthritis. Pharmacological Research.

[43] Kamal FA, Travers JG, Blaxall BC (2012). G protein-coupled receptor kinases in cardiovascular disease: why "where" matters. Trends Cardiovasc Med.

[44] Carlson EL, Karuppagounder V, Pinamont WJ, Yoshioka NK, Ahmad A, Schott EM (2021). Paroxetine-mediated GRK2 inhibition is a disease-modifying treatment for osteoarthritis. Sci Transl Med.

