# Supplementary material for: Inhibition of GRK2-PDE4D Axis Suppresses Fibroblast-Like Synoviocytes Hyperplasia and Alleviates Experimental Arthritis
Source: Int J Biol Sci. 2025 Jan 27;21(4):1513–29. doi: 10.7150/ijbs.100176 (PMC11844291; doi:10.7150/ijbs.100176)
Supplement: Supplementary file 1 — Supplementary figures and tables. [file ijbsv21p1513s1.pdf]

Supplementary Information for

Inhibition of GRK2-PDE4D Axis Suppresses Fibroblast-Like Synoviocytes Hyperplasia and Alleviates Experimental Arthritis

Dafei Han[1#], Hanfei Sun[1#], Renhao Zhang[1#], Hui Ge[1], Paipai Guo[1], Rui Chu[1], Ruhong Fang[1], Yongsheng Han[2], Shufang He[3], Rui Li[3], Jiajie Tu[1], Wei Wei[1*], Yang Ma[1*], Qingtong Wang[1, 4*]

1 Institute of Clinical Pharmacology, Anhui Medical University, Key Laboratory of Anti-Inflammatory and Immune Medicine, Ministry of Education, Anhui Collaborative Innovation Center of Anti-Inflammatory and Immune Medicine, Hefei, 230032, China.

2 Department of Emergency Medicine, The First Affiliated Hospital of USTC, Division of Life Sciences and Medicine, University of Science and Technology of China, Hefei, 230001, China.

3 Department of Anesthesiology and Perioperative Medicine, the Second Hospital of Anhui Medical University, Hefei 230601, China.

4 The Third Affiliated Hospital of Anhui Medical University (The First People's Hospital of Hefei), Hefei 230061, China.

This Microsoft Word file includes:

Supplemental Figures and Figure Legends (Supplementary Figure 1 to 5)

Supplementary Tables S1 to S2

Supplementary Figures and Figure Legends

Supplementary Figure 1

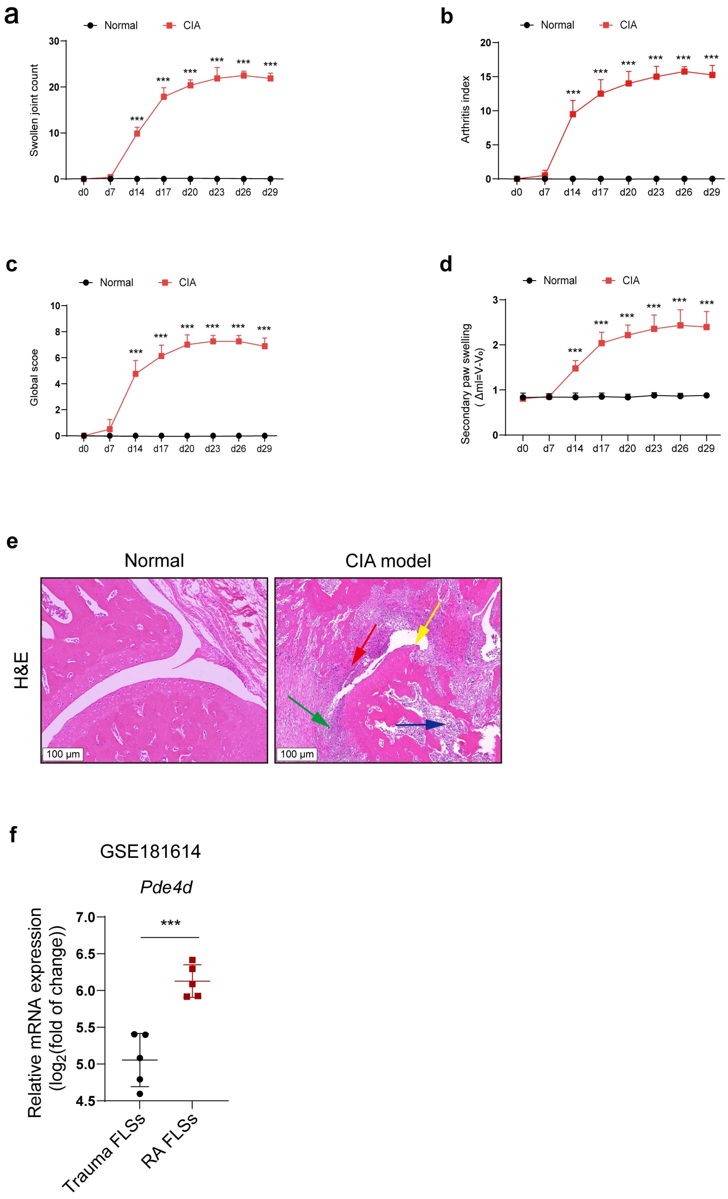

Supplementary Figure 1. The rat CIA model was successfully established. (a) The swollen joint count, (b) arthritis index, (c) global score, and (d) secondary paw

swelling of rats were recorded from the 0th day to the 29th day after the first immunization. Data are presented as mean ± SD. $^{***}p < 0.001$ by Two-way ANOVA followed by Tukey's multiple comparisons test, n = 6. (e) Representative photographs of H&E staining of the knee joint sections from normal and CIA rats. Pathological changes include synovial hyperplasia (red arrowhead), pannus (blue arrowhead), infiltrating inflammatory cells (green arrowhead), and cartilage destruction (yellow arrowhead). (f) RNA Seq analysis (GSE181614) on FLSs from trauma and RA patients revealed significantly increased levels of *Pde4d* mRNA in RA patients compared with trauma patients. Data are presented as mean ± SD. $^{***}p < 0.001$ by unpaired t-test, n = 5.

Supplementary Figure 2

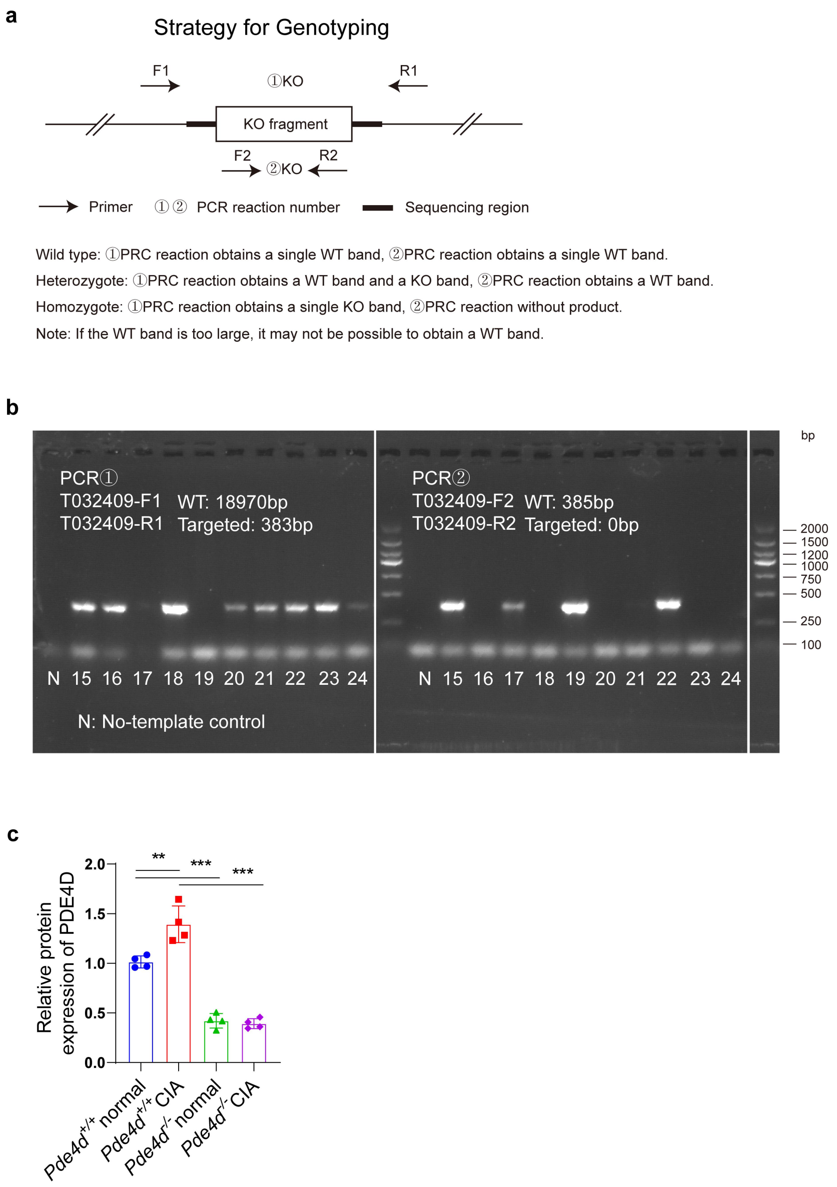

Supplementary Figure 2. *Pde4d*$^{-/-}$ mice were successfully constructed. (b) Strategy of genotyping for *Pde4d*$^{-/-}$ mice. (b) Representative images of genotyping. (c) Quantification of PDE4D protein expression levels from hind paws of different

groups. Data are presented as mean ± SD. $^{**}p < 0.01$ and $^{***}p < 0.001$ by two-way ANOVA followed by Tukey's multiple comparisons test, n = 4.

Supplementary Figure 3

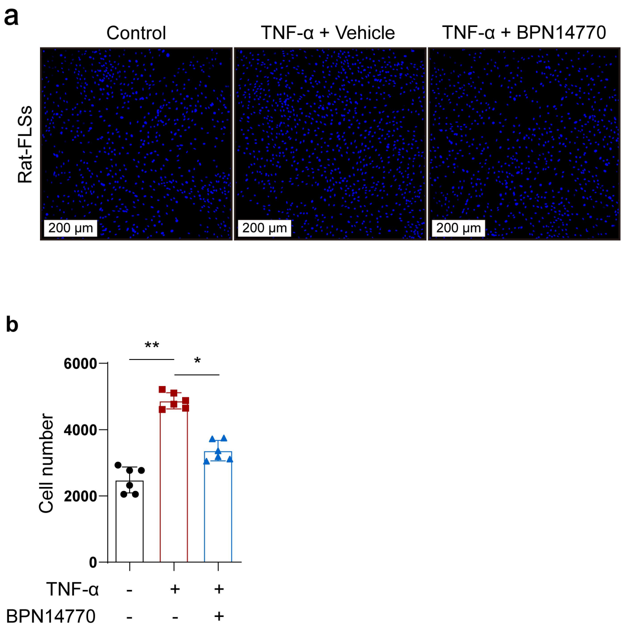

Supplementary Figure 3. Inhibition of PDE4D significantly suppressed TNF-α-induced FLSs proliferation. (a) Representative images of cell number. (b) Quantification of cell number by HCCIS. Data are presented as mean ± SD. $^{*}p < 0.05$

and $^{**}p < 0.01$ by two-way ANOVA followed by Tukey's multiple comparisons test, n = 5.

Supplementary Figure 4

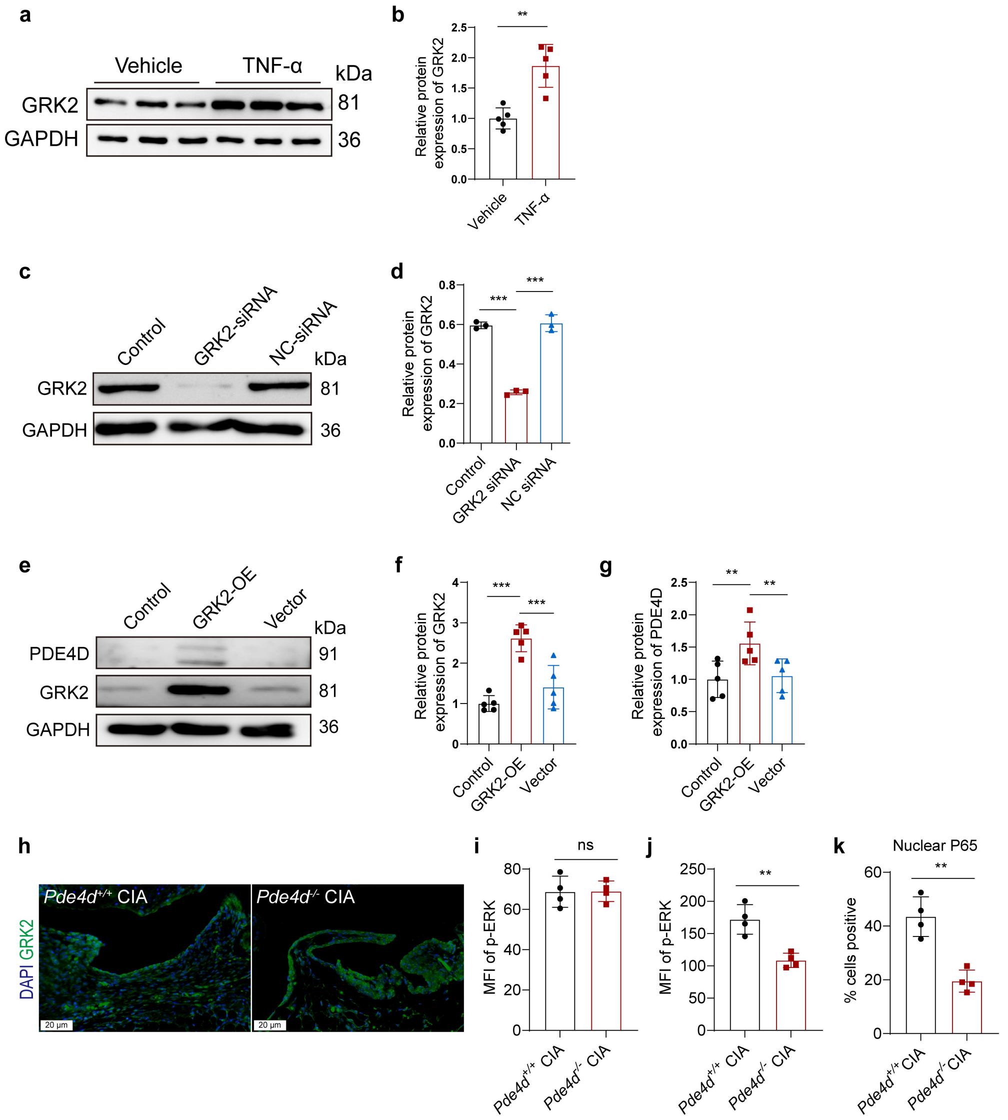

Supplementary Figure 4. Overexpressed PDE4D in TNF-α-treated FLS was mediated by GRK2. (a) Immunoblot analysis and (b) quantification of GRK2 protein expression in normal rats FLS treated with TNF-α or vehicle. Data are presented as mean ± SD. $^{**}p < 0.01$ by unpaired t test, n = 5. (c) Immunoblot analysis and (d) quantification of GRK2 protein expression in control, GRK2 siRNA and NC-siRNA FLS. Data are presented as mean ± SD. $^{***}p < 0.001$ by two-way ANOVA followed by Tukey's multiple comparisons test, n = 3. (e) Immunoblot analysis and (f and g) quantification of GRK2 and PDE4D protein expression in control, GRK2-OE and vector FLS. Data are presented as mean ± SD. $^{**}p < 0.01$ and $^{***}p < 0.001$ by two-way ANOVA followed by Tukey's multiple comparisons test, n = 5. (h) Representative GRK2 IF staining and (i) the mean fluorescence intensity (MFI) of GRK2 in the synovium of CIA mice. n = 4. Data are presented as mean ± SD. ns, no significance. (j) The mean fluorescence intensity (MFI) of p-ERK and (k) the percentage of nuclear P65-positive cells in the synovium of CIA mice. n = 4. Data are presented as mean ± SD. $^{**}p < 0.01$ by unpaired t test.

Supplementary Figure 5

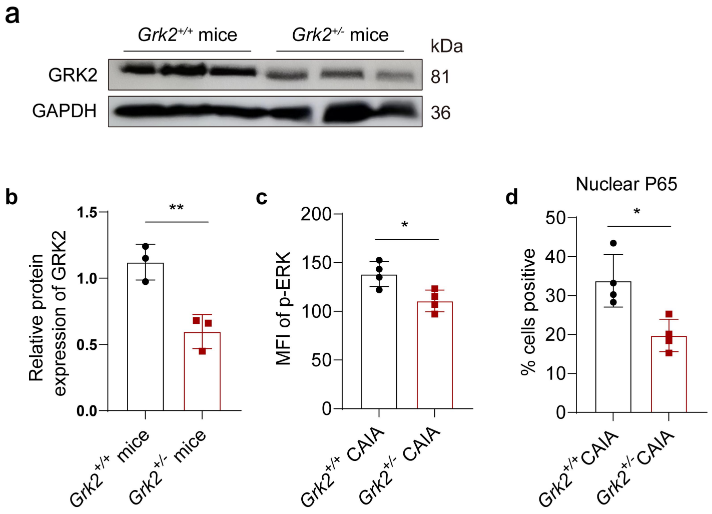

Supplementary Figure 5. The knock down efficiency of GRK2 in *Grk2*$^{+/-}$ mice. (a) Immunoblot analysis GRK2 protein expression levels in *Grk2*$^{+/+}$ and *Grk2*$^{+/-}$ mice. (b) Quantification of GRK2 protein expression in *Grk2*$^{+/+}$ and *Grk2*$^{+/-}$ mice. Data are

presented as mean ± SD. $^{**}p < 0.01$ by unpaired t test, n = 3. (c) The mean fluorescence intensity (MFI) of p-ERK and (d) the percentage of nuclear P65-positive cells in the synovium of CAIA mice. n = 4. Data are presented as mean ± SD. $^{*}p < 0.05$ by unpaired t test.

Supplementary Tables

Table S1. The specific sequences and primers used in this investigation.

Name	Primer sequences (5'-3')	
	Forward	Reverse
Pde4a	GTGGAGAAGTCTCAGGTGGG	TGGAACTTGTCAGGCAGGG
Pde4b	GATGAGCAGATCAGGGAACC	GATGGGATTTCCACATCGTT
Pde4c	GACCCTGTCCTTCCTGTTGA	AACCGTCTCAGGATCACACC
Pde4d	GCCAGCCTTCGAACTGTAAG	ATGGATGGTTGGTTGCACAT
NC siRNA	UUCUCCGAACGUGUCACGUTT	ACGUGACACGUUCGGAGAATT
GRK2 siRNA	CCAUGAAGUGUCUGGACAATT	UUGUCCAGACACUUGAUGGTT
PCR①	GCATGAAGGATACTTCCCAAGACTG	GACTGGGGTGCCTCTGAGCTTTTA
PCR②	GAACAGACATAGAGCACCTTCCACA	CCTTGAGCCTGTTAGTGCTGGGAT

Table S2. The sources of antibodies used in this study.

Antibodies	Catalogue number	Company	Location
Primary antibodies			
Anti-p65	sc-8008	Santa Cruz Biotechnology	Santa Cruz, CA, USA
Anti-vimentin	sc-6260	Santa Cruz Biotechnology	Santa Cruz, CA, USA
Anti-GRK2	sc-13143	Santa Cruz Biotechnology	Santa Cruz, CA, USA
Anti-p-p65	3033	Cell Signaling Technology	Danvers, MA, USA
Anti-ERK	4696	Cell Signaling Technology	Danvers, MA, USA
Anti-p-ERK	4370	Cell Signaling Technology	Danvers, MA, USA
Anti-p38	8690	Cell Signaling Technology	Danvers, MA, USA
Anti-p-p38	9216	Cell Signaling Technology	Danvers, MA, USA
Anti-PDE4D	12918-1-AP	Proteintech	Chicago, IL, USA
Anti-GAPDH	60004-1-Ig	Proteintech	Chicago, IL, USA
Secondary antibodies			
Alexa Fluor 488-Anti-mouse	115-545-003	Jackson Immuno Research Laboratories	West Grove, PA, USA
Alexa Fluor 594-Anti-rabbit	11-585-003	Jackson Immuno Research Laboratories	West Grove, PA, USA
